# Subject clustering by IF-PCA and several recent methods

**DOI:** 10.3389/fgene.2023.1166404

**Published:** 2023-05-23

**Authors:** Dieyi Chen, Jiashun Jin, Zheng Tracy Ke

**Affiliations:** ^1^ Department of Statistics, Harvard University, Cambridge, MA, United States; ^2^ Department of Statistics, Carnegie Mellon University, Pittsburgh, PA, United States

**Keywords:** gene microarray, feature selection, higher criticism threshold, PCA, ScRNA-seq, sparsity, subject clustering, variational

## Abstract

Subject clustering (i.e., the use of measured features to cluster subjects, such as patients or cells, into multiple groups) is a problem of significant interest. In recent years, many approaches have been proposed, among which unsupervised deep learning (UDL) has received much attention. Two interesting questions are 1) how to combine the strengths of UDL and other approaches and 2) how these approaches compare to each other. We combine the variational auto-encoder (VAE), a popular UDL approach, with the recent idea of influential feature-principal component analysis (IF-PCA) and propose IF-VAE as a new method for subject clustering. We study IF-VAE and compare it with several other methods (including IF-PCA, VAE, Seurat, and SC3) on 10 gene microarray data sets and eight single-cell RNA-seq data sets. We find that IF-VAE shows significant improvement over VAE, but still underperforms compared to IF-PCA. We also find that IF-PCA is quite competitive, slightly outperforming Seurat and SC3 over the eight single-cell data sets. IF-PCA is conceptually simple and permits delicate analysis. We demonstrate that IF-PCA is capable of achieving phase transition in a rare/weak model. Comparatively, Seurat and SC3 are more complex and theoretically difficult to analyze (for these reasons, their optimality remains unclear).

## 1 Introduction

We are interested in the problem of *high-dimensional clustering* or *subject clustering*. Suppose we have a group of *n* subjects (e.g., patients or cells) measured on the same set of *p* features (e.g., genes). The subjects come from *K* different classes or groups (e.g., normal group and diseased group), but unfortunately, the class labels are unknown. In such a case, we say the data are *unlabeled*. For 1 ≤ *i* ≤ *n*, we denote the class label of subject *i* by *Y*_*i*_ and denote the *p*-dimensional measured feature vector of subject *i* by *X*_*i*_. It is important to note that *Y*_*i*_ takes values from {1, 2, …, *K*}. The class labels are unknown, and the goal is to predict them using the measured features *X*_1_, *X*_2_, …, *X*_*n*_.

High-dimensional clustering is an unsupervised learning problem. It is especially interesting in the *big data era*: although the volume of available scientific data increases rapidly, a significant fraction of them are unlabeled. In some cases, it is simply hard to label each individual sample [e.g., action unit recognition (Wu et al., 2015)]. In some other cases, labeling each individual sample is not hard, but due to the large sample size, it takes a substantial amount of time and effort to label the whole data set [e.g., ImageNet (Deng et al., 2009)]. In other instances (e.g., cancer diagnosis), we may have a preliminary opinion on how to label the data, but we are unsure of the labels’ accuracy, so we would like a second, preferably independent, opinion. In all these cases, we seek an effective and user-friendly clustering method.

In recent years, the area of high-dimensional clustering has witnessed exciting advancements in several directions. First, many new types of data sets (e.g., single-cell data) have emerged and become increasingly more accessible. Second, remarkable successes have been made in non-linear modeling for high-dimensional data, and several unsupervised deep learning (UDL) approaches have been proposed (Fan et al., 2021), including, but not limited to, the variational auto-encoder (VAE) and generative adversarial network (GAN). Finally, several clustering methods for single-cell data [e.g., Seurat (Satija et al., 2015) and SC3 (Kiselev et al., 2017)] have been proposed and become popular.

In this paper, we are primarily interested in influential feature-principal component analysis (IF-PCA), a clustering algorithm proposed by Jin and Wang (2016). As in many recent works in high-dimensional data analysis (e.g., Abramovich et al., 2006; Paul, 2007), we assume

• *p* ≫ *n* ≫ 1
• Out of all *p* measured features, only a small fraction of them are relevant to the clustering decision.

IF-PCA is easy-to-use and does not have tuning parameters. It is conceptually simple and (on a high level) contains two steps as follows:

• IF step*.* A feature selection step that selects a small fraction of measured features, which we believe to be influential or significant to the clustering decision.
• Clustering step*.* A clustering step in which PCA (as a spectral clustering approach) is applied to all retained features.

Instead of viewing IF-PCA as a specific clustering algorithm, we can view it as a *generic two-step clustering approach*: for each of the two steps, we can choose methods that may vary from occasion to occasion in order to best suit the nature of the data. We anticipate that IF-PCA will adapt and develop over time as new data sets and tasks emerge.

Jin and Wang (2016) compared IF-PCA to a number of clustering algorithms [including the classical k-means (MacQueen, 1967), k-means++ (Arthur and Vassilvitskii, 2007), Spectral-Gem (Lee et al., 2010b), hierarchical clustering (Hastie et al., 2009), and sparse PCA (Zou et al., 2006)] using 10 microarray data sets. They found that IF-PCA was competitive in clustering accuracy. Later, Jin et al. (2017) developed a theoretical framework for clustering and showed that IF-PCA is optimal in the rare/weak signal model [a frequently used model in high-dimensional data analysis (Donoho and Jin, 2004; Donoho and Jin, 2015)].

These appealing properties of IF-PCA motivate a revisit of this method. Specifically, we are interested in the two questions listed below.

• There are many recent clustering algorithms specifically designed for single-cell data, such as Seurat (Satija et al., 2015), SC3 (Kiselev et al., 2017), RaceID (Grün et al., 2015), ActioNet (Mohammadi et al., 2020), Monocle 3 (Trapnell et al., 2014), and SINCERA (Guo et al., 2015). In addition, many UDL algorithms have been proposed and become well-known in recent years. An interesting question is how IF-PCA compares with these popular algorithms.

Jin and Wang (2016) only examined IF-PCA with gene microarray data. The single-cell RNA-seq data are similar to gene microarray data in some aspects but also have some distinguished characteristics [e.g., single-cell RNA-sequencing provides an unbiased view of all transcripts and is, therefore, reliable for accurately measuring gene expression level changes (Zhao et al., 2014)]. How IF-PCA compares to other popular methods for subject clustering with single-cell data is an intriguing question.

• The PCA employed in the clustering step of IF-PCA is a linear method. Although we believe that the associations between class labels and measured features may be non-linear, the significance of the non-linear effects is unclear. To investigate this, we may consider a variant of IF-PCA, in which PCA is replaced by some non-linear UDL methods in the clustering step. An interesting question is how this variant compares to IF-PCA and standard UDL methods (which has no IF step). It helps us understand how significant the non-linear effects are.

To answer these questions, first, we propose a new approach, IF-VAE, which combines the main idea of IF-PCA with the VAE (Kingma and Welling, 2013) (one of the most popular UDL approaches in recent literature).

Second, we compare the IF-VAE with several methods, including VAE, IF-PCA, Spectral-Gem (Lee et al., 2010b), and classical k-means, using the 10 microarray data sets in Jin and Wang (2016). We find that

• Somewhat surprisingly, the VAE underperforms compared to most other methods, including the classical k-means.
• IF-VAE, which combines the VAE with the IF step of IF-PCA, significantly outperforms the VAE.
• The performance of IF-PCA and IF-VAE is comparable for approximately half of the data sets, whereas IF-VAE significantly underperforms compared to IF-PCA for the remaining half of the data sets.

These results suggest the following:

• (a) The idea of combining the IF step in the IF-PCA with the VAE is valuable.
• (b) Deep neural network methods do not appear to have a clear advantage for this type of data set.

For (b), one possible reason is that the associations between class labels and measured features are not highly non-linear. Another possible reason is that existing deep neural network approaches need further improvements in order to perform satisfactorily on these data sets. Since IF-PCA and IF-VAE use the same IF step, the unsatisfactory performance of IF-VAE is largely attributable to the VAE step, and not the IF step. To see this, we observe that Spectral-Gem is essentially the classical PCA clustering method (see Section 2.2). The VAE does not show an advantage over Spectral-Gem, explaining why IF-VAE cannot outperform IF-PCA.

Last, we compare the IF-VAE with IF-PCA, Seurat, and SC3 on eight single-cell RNA-seq data sets. We observe that

• IF-VAE continues to underperform compared to other methods on the eight single-cell data sets, but similar as previously mentioned; the unsatisfactory performance is largely attributable to the VAE step and not the IF step.
• IF-PCA outperforms SC3 slightly and outperforms Seurat more significantly.

At the same time, we observe that

• Seurat has four tuning parameters and is the method that has the shortest execution time.
• The idea of SC3 is quite similar to that of IF-PCA, except that SC3 has a “consensus voting” step that aggregates the strengths of many clustering results. With consensus voting, SC3 may empirically perform more satisfactorily, but it is also more complex internally. Regarding the computational cost, it runs much slower than IF-PCA due to the consensus voting step.

Moreover, IF-PCA is conceptually simple and permits fine-grained analysis. In Section 4, we develop a theoretical framework and show that IF-PCA achieves the optimal phase transition in a rare/weak signal setting. Especially, we show in the region of interest (where successful subject clustering is possible),

• if the signals are less sparse, signals may be individually weak. In this case, PCA is optimal (and IF-PCA reduces to PCA if we choose the IF step properly).
• If the signals are sparser, the signals need to be relatively strong (so successful clustering is possible). In this case, feature selection is necessary and IF-PCA is optimal. However, PCA may be non-optimal as it does not use a feature selection step.

In comparison, other popular methods are difficult to analyze theoretically; hence, their optimality is unclear. We note that hard-to-analyze methods will also be hard to improve in the future.

In conclusion, IF-PCA is quite competitive compared to the recently popular subject clustering methods, both for gene microarray data and single-cell data. It is worthwhile to study IF-PCA both theoretically and in (a variety of) applications. IF-VAE is a significant improvement over VAE, but it is still inferior to other prevalent methods in this area (the underperformance is largely due to the VAE step, not the IF step). It is desirable to further improve IF-VAE (especially the VAE step) to make it more competitive.

## 2 Models and methods

As before, suppose we have measurements on the same set of *p* features for *n* samples. We denote the data matrix by $X\in R^{n,p}$ and write

$$X={\left[X_{1},X_{2},\ldots ,X_{n}\right]}^{\prime }=\left[x_{1},x_{2},\ldots ,x_{p}\right],$$ (1)

where $X_{i}\in R^{p}$ denotes the measured feature vector for sample *i*, 1 ≤ *i* ≤ *n*. From time to time, we may want to normalize the data matrix before we implement any approaches. For 1 ≤ *j* ≤ *p*, let $\hat{X}\left(j\right)$ and $\hat{\sigma }\left(j\right)$ be the empirical mean and standard deviation associated with feature *j* (column *j* of *X*), respectively. We normalize each column of *X* and denote the resultant matrix by *W*, where

$$W=\left[w_{1},w_{2},\ldots ,w_{p}\right]={\left[W_{1},W_{2},\ldots W_{n}\right]}^{\prime }\in R^{n,p},\;\;\;\text{and}\;\;\;W_{i}\left(j\right)=\left[X_{i}\left(j\right)-\hat{X}\left(j\right)\right]/\hat{\sigma }\left(j\right).$$ (2)

In Section 2.1, we introduce two models for *X*; then in Sections 2.2, 2.3, 2.4, 2.5, 2.6, we describe the clustering methods considered in this paper, some of which (e.g., IF-VAE, IF-VAE(X), and IF-PCA(X)) are new.

### 2.1 Two models

A reasonable model is as follows. We encode the class label *Y*_*i*_ as a *K*-dimensional vector *π*_*i*_, where *π*_*i*_ = *e*_*k*_ if and only if sample *i* belongs to class *k*, and *e*_*k*_ is the *k*th standard Euclidean basis vector of $R^{K}$ , 1 ≤ *k* ≤ *K*. Let *M* = [*μ*_1_, *μ*_2_, … , *μ*_*K*_], where $\mu_{k}\in R^{p}$ is the mean vector for class *k*. We assume

$$E\left[X_{i}\right]=\mu_{k}\text{ if and only if subject}\;\;i\;\;\text{belongs to class}\;\;k,\;\text{or equivalently}\;\;E\left[X_{i}\right]=M\pi_{i}.$$ (3)

Let $\Pi ={\left[\pi_{1},\pi_{2},\ldots ,\pi_{n}\right]}^{\prime }$ be the matrix of encoded class labels. We can rewrite (3) as

$$X=E\left[X\right]+\left(X-E\left[X\right]\right)=“\text{signal matrix}”+“\text{noise matrix}”,\;E\left[X\right]=\Pi M^{\prime }.$$ (4)

In addition, it is reasonable to assume that out of many measured features, only a small fraction of them are useful in the clustering decision. Therefore, letting $\bar{\mu }=\left(1/K\right)\sum_{k=1}^{K}\mu_{k}$ , we assume

$$\mu_{1},\mu_{2},\ldots ,\mu_{K}\;\;\text{are linearly independent and}\;\;\mu_{k}-\bar{\mu }\;\;\text{is sparse for each}\;\;1\leq k\leq K.$$ (5)

It follows that the *n* × *p* signal matrix E[X] has a rank *K*.

We recall that *W* is the normalized data matrix. Similar to (5), we may decompose *W* as the sum of a signal matrix and a noise matrix. However, due to the normalization, the rank of the signal matrix is reduced to (*K* − 1).

In Models (3)–(5), $E\left[X_{i}\right]=M\pi_{i}$ , which is a linear function of the encoded class label vectors *π*_*i*_. For this reason, we may view Models (3)–(5) as linear models. In many modern applications, linear models may be inadequate, and we may prefer to use a non-linear model.

The recent idea of neural network modeling provides a wide class of non-linear models, which may be useful for our setting. As an alternative to Models (3)–(5), we may consider a neural network model as follows. In this model, we assume

$$Y_{i}=f\left(X_{i},\theta \right),\;i=1,2,\ldots ,n,$$ (6)

where *f* (*x*, *θ*) belongs to a class of non-linear functions. For example, we may assume *f* (*x*, *θ*) belongs to the class of functions (without loss of generality, *x* always includes a constant feature):

$$\left\{f\left(x,\theta \right):f\left(x,\theta \right)=A_{L}\left(s_{L}\left(\right.A_{L-1}\ldots s_{2}\left(A_{2}s_{1}\left(A_{1}x\right)\right)\right)|\theta =\left\{A_{1},A_{2},\ldots ,A_{L}\right\}\right\},$$

where *A*_1_, *A*_2_, … , *A*_*L*_ are matrices of certain sizes and *s*_1_, *s*_2_, … , *s*_*L*_ are some nonlinear functions. Similar to Models (3)–(5), we can impose some sparsity conditions on Model (6). See Fan et al. (2021) for example.

### 2.2 The PCA clustering approach and Spectral-Gem

PCA is a classical spectral clustering approach, which is especially appropriate for linear models like those in (3–5) when the relevant features are non-sparse (see the following text for discussions on the case when the relevant features are sparse). The PCA clustering approach contains two simple steps as follows. Input: normalized data matrix *X* and number of clusters *K*. Output: predicted class label vector $\hat{Y}={\left({\hat{Y}}_{1},{\hat{Y}}_{2},\ldots ,{\hat{Y}}_{n}\right)}^{\prime }$ .

• We obtain the *n* × *K* matrix $\hat{H}=\left[{\hat{\eta }}_{1},\ldots ,{\hat{\eta }}_{K}\right]$ , where ${\hat{\eta }}_{k}$ is the *k*th left singular vector of *X* (associated with the *k*th largest singular value of *X*).
• We cluster the *n* rows of $\hat{H}$ to *K* groups by applying the classical k-means assuming there are ≤K classes. Let ${\hat{Y}}_{i}$ be the estimated class label of subject *i*. Output ${\hat{Y}}_{1},\ldots ,{\hat{Y}}_{n}$ .

From time to time, we choose to apply the PCA clustering approach to the normalized data matrix *W*. As explained before, we can similarly write *W* as the sum of a “signal” matrix and a “noise” matrix as in (5), but due to the normalization, the rank of the “signal” matrix under Model (3) is reduced from *K* to (*K* − 1). In such a case, we replace the *n* × *K* matrix $\hat{H}$ by the *n* × (*K* − 1) matrix

$$\hat{\Xi }=\left[{\hat{\xi }}_{1},{\hat{\xi }}_{2},\ldots ,{\hat{\xi }}_{K-1}\right],$$

where, similarly, ${\hat{\xi }}_{k}$ is the *k*th left singular vector of *W*.

The PCA clustering approach has many modern variants, including, but not limited to, Spectral-Gem (Lee et al., 2010b) and SCORE (Jin, 2015; Ke and Jin, 2023). In this paper, we consider Spectral-Gem but skip the discussion on SCORE (SCORE was motivated by unsupervised learning in network and text data and shown to be effective on those types of data; it is unclear whether SCORE is also effective for genetic and genomic data). Instead of applying PCA clustering to the data matrix *X* (or *W*) directly, Spectral-Gem constructs an *n* × *n* symmetric matrix *M*, where *M*(*i*, *j*) can be viewed as a similarity metric between subject *i* and subject *j*. The remaining part of the algorithm has many small steps, but the essence is to apply the PCA clustering approach to the Laplacian normalized graph induced by *M*.

The PCA spectral clustering approach is based on two important assumptions.

• The signal matrix E[X] is a linear function of class labels.
• It is hard to exploit sparsity in the data: either the data are non-sparse (such as the classical setting of *p* ≪ *n*) or how the sparsity can be exploited is unclear.

In many modern settings, these assumptions are not satisfied: the relationship between the signal matrix E[X] and class labels may be non-linear, and it is highly desirable to exploit sparsity by adding a feature selection before conducting PCA clustering. In such cases, we need an alternative approach. We address the non-linearity by the VAE and feature selection by IF-PCA as follows.

### 2.3 The variational autoencoder and VAE(X) clustering approaches

Given an *n* × *p* data matrix *X* and an integer *d* ≤ rank(*X*), the essence of the PCA spectral clustering approach is to obtain a rank-*d* approximation of *X* to use singular value decomposition (SVD),

$$\hat{X}=\overset{d}{\underset{k=1}{\sum }}\sigma_{k}u_{k}v_{k}^{\prime }.$$

Here, *σ*_*k*_ is the *k*th smallest singular value of *X*, and *u*_*k*_ and *v*_*k*_ are the corresponding left and right singular vectors of *X*, respectively. The VAE can be viewed as an extension of SVD, which obtains a rank-*d* approximation of *X* from training a neural network. The classical SVD is a linear method, but the neural network approach can be highly non-linear.

The VAE was first introduced by Kingma and Welling (2013) and has been successfully applied to many areas [e.g., image processing (Razavi et al., 2019), computer vision (Goodfellow et al., 2020), and text mining (Serban et al., 2017)]. The VAE consists of an encoder, a decoder, and a loss function. Given a data matrix $X\in R^{n,p}$ , the encoder embeds *X* into a matrix $\hat{Z}\in R^{n,d}$ (usually *d* ≪ *p*), and the decoder maps $\hat{Z}$ back to the original data space and outputs a matrix $\hat{X}\in R^{n,p}$ , which can be viewed as a rank-*d* approximation of *X*. Different from classical SVD, $\hat{X}$ is obtained in a non-linear fashion by minimizing an objective that measures the information loss between *X* and $\hat{X}$ .

A popular way to use the VAE for subject clustering is as follows (Wang and Gu, 2018). Input: normalized data matrix $W=\left[w_{1},w_{2},\ldots ,w_{p}\right]={\left[W_{1},W_{2},\ldots ,W_{n}\right]}^{\prime }$ , number of classes *K*, and dimension of the latent space *d* (typically much smaller than min{*n*, *p*}). Output: predicted class label vector $\hat{Y}=\left({\hat{Y}}_{1},{\hat{Y}}_{2},\ldots ,{\hat{Y}}_{n}\right)$ .

• (Dimension reduction by the VAE). We train the VAE and use the trained encoder to obtain an *n* × *d* matrix $\hat{Z}$ .
• (Clustering). We cluster all *n* subjects into *K* classes by applying k-means to the rows of $\hat{Z}$ . Let $\hat{Y}$ be the predicted label vector.

Except for using a non-linear approach to dimension reduction, the VAE is similar to the PCA approach in clustering. We can apply the VAE either to the normalized data matrix *W* or the unnormalized data matrix *X*. We call them VAE(W) and VAE(X), respectively. In the context of using these notations, it is unnecessary to use (W) and (X) at the same time, so we write VAE(W) as VAE for short (and to avoid confusion, we still write VAE(X) as VAE(X)).

### 2.4 The orthodox IF-PCA and its variant IF-PCA(X)

For many genomic and genetic data, Models (3)–(5) are already reasonable models. We recall that under these models, the normalized data matrix can be approximately written as

$$W=Q+\left(W-Q\right)=“\text{signal matrix}”+“\text{noise matrix}”,$$

where, approximately,

$$Q=\Pi {\left[\mu_{1}-\bar{\mu },\mu_{2}-\bar{\mu },\ldots ,\mu_{K}-\bar{\mu }\right]}^{\prime }\in R^{n,p},$$

and is sparse (in the sense that only a small fraction of the columns of *Q* have a large *ℓ* ^2^-norm; the *ℓ* ^2^-norm of other columns is small or 0). In such a setting, it is appropriate to conduct feature selection, which removes a substantial amount of noise while keeping most non-zero columns of *Q*.

Such observations motivate the (orthodox) IF-PCA. The IF-PCA was first proposed in Jin and Wang (2016) and has been shown to have appealing clustering results with 10 gene microarray data sets. In Jin et al. (2017), it was shown that IF-PCA is optimal in high-dimensional clustering. IF-PCA contains an IF step and a PCA step, and the IF step contains two important components introduced as follows.

The first component of the IF step is the use of the Kolmogorov–Smirnov (KS) test for feature selection. Suppose we have *n* (univariate) samples *z*_1_, *z*_2_, … , *z*_*n*_ from a cumulative distribution function (CDF) denoted by *F*. We introduce the empirical CDF by

$$F_{n}\left(t\right)=\left(1/n\right)\overset{n}{\underset{i=1}{\sum }}1\left\{z_{i}\leq t\right\}.$$ (7)

Let *z* = (*z*_1_, *z*_2_, … , *z*_*n*_). The KS testing score is then

$$\phi_{n}\left(z\right)=\sqrt{n}\sup_{t}\left\{\left\|F_{n}\left(t\right)-F\left(t\right)\right\|\right\}.$$ (8)

In the IF-PCA given as follows, we take *F* to be the theoretical CDF of $\left(z_{i}-\bar{z}\right)/\hat{\sigma }$ , where $z_{i}\overset{iid}{∼}N\left(0,1\right)$ ; 1 ≤ *i* ≤ *n*; and $\bar{z}$ and $\hat{\sigma }$ are the empirical mean and standard deviation of *z*_1_, *z*_2_, … , *z*_*n*_, respectively.

The second component of the IF step is the higher criticism threshold (HCT). Higher criticism (HC) was initially introduced by Donoho and Jin (2004) (see also Jager and Wellner, 2007; Hall and Jin, 2010; Donoho and Jin, 2015; Verzelen and Arias-Castro, 2017) as a method for global testing. It has been recently applied to genetic data (e.g., Barnett et al., 2017). HCT adapts HC to a data-driven threshold choice (Jin and Wang, 2016). It takes as input *p* marginal *p*-values, one for a feature, and outputs a threshold for feature selection. Suppose we have *p*-values *π*_1_, *π*_2_, … , *π*_*p*_. We sort them in the ascending order:

$$\pi_{\left(1\right)}<\pi_{\left(2\right)}<\ldots <\pi_{\left(p\right)}.$$

We define the feature-wise HC score by $HC_{p,j}=\sqrt{p}\left(j/p-\pi_{\left(j\right)}\right)/\sqrt{\max\left\{\sqrt{n}\left(j/p-\pi_{\left(j\right)}\right),0\right\}+j/p}$ . The HCT is then

$${\hat{t}}_{HC}=\pi_{\left(\hat{j}\right)},\;\text{where}\;\;\hat{j}={argmax}_{\left\{j:\pi_{\left(j\right)}>\log p/p,\;j<p/2\right\}}\left\{HC_{p,j}\right\}.$$ (9)

IF-PCA runs as follows. Input: normalized feature vectors $W=\left[w_{1},w_{2},\ldots ,w_{p}\right]={\left[W_{1},W_{2},\ldots ,W_{n}\right]}^{\prime }$ and number of classes *K*. Output: predicted class label vector $\hat{Y}={\left({\hat{Y}}_{1},{\hat{Y}}_{2},\ldots ,{\hat{Y}}_{n}\right)}^{\prime }$ .

• (IF step). For each 1 ≤ *j* ≤ *p*, we compute a KS score for feature *j* by applying (7, 8) with *z* = *w*_*j*_. We denote the KS scores by *ϕ*_*n*_ (*w*_1_), …, *ϕ*_*n*_ (*w*_*p*_) and let μ* and σ* be their empirical mean and standard deviation, respectively. Let $\psi_{j}^{*}=\left[\phi_{n}\left(w_{j}\right)-\mu^{*}\right]/\sigma^{*}$ . We compute the *p*-values by $\pi_{j}=1-F\left(\psi_{j}^{*}\right)$ , where *F* is the same CDF used in (8). We obtain the HCT by applying (9) to *π*_1_, *π*_2_, … , *π*_*p*_. We retain feature *j* if $\pi_{j}\leq {\hat{t}}_{HC}$ and remove it otherwise.
• (Clustering step). Let *W* ^*IF*^ be the *n* × *m* sub-matrix of *W* consisting of columns of *W* corresponding to the retained features only [*m* is the number of retained features in (a)]. For any 1 ≤ *k* ≤ min{*m*, *n*}, let ${\hat{\xi }}_{k}^{IF}$ be the left singular vector of *W* ^*IF*^ corresponding to the *k*th largest singular value of *W* ^*IF*^. Let ${\hat{\Xi }}^{IF}=\left[{\hat{\xi }}_{1}^{IF},\ldots ,{\hat{\xi }}_{K-1}^{IF}\right]\in R^{n,K-1}$ . We cluster all *n* subjects by applying the *k*-means to the *n* rows of ${\hat{\Xi }}^{IF}$ , assuming there are *K* clusters. Let $\hat{Y}={\left({\hat{Y}}_{1},{\hat{Y}}_{2},\ldots ,{\hat{Y}}_{n}\right)}^{\prime }$ be the predicted class labels.

In the IF step, the normalization of $\psi_{j}^{*}=\left[\phi_{n}\left(w_{j}\right)-\mu^{*}\right]/\sigma^{*}$ is called Efron’s null correction (Efron, 2004), a simple idea that is proven to be both necessary and effective for analyzing genomic and genetic data (Jin et al., 2015). We remark that although IF-PCA is motivated by the linear model in (5), it is not tied to (5) and is broadly applicable. In fact, the algorithm does not require any knowledge of Models (3)–(5).

In the (orthodox) IF-PCA, we apply both the IF step and the clustering step to the normalized data matrix *W*. Seemingly, for the IF step, applying the algorithm to *W* instead of the un-normalized data matrix *X* is preferred. However, for the clustering step, whether we should apply the algorithm to *W* or *X* remains unclear. We propose a small variant of IF-PCA by applying the IF step and the clustering step to *W* and *X*, respectively.

• (IF step). We apply exactly the same IF step to *W* as in the (orthodox) IF-PCA previously mentioned.
• (Clustering step). Let *X* ^*IF*^ be the *n* × *m* sub-matrix of *X* consisting of columns of *X* corresponding to the retained features in the IF step only. For any 1 ≤ *k* ≤ min{*m*, *n*}, let ${\hat{\eta }}_{k}^{IF}$ be the left singular vector of *X* ^*IF*^ corresponding to the *k*th largest singular value of *X* ^*IF*^. Let ${\hat{H}}^{IF}=\left[{\hat{\eta }}_{1}^{IF},\ldots ,{\hat{\eta }}_{K-1}^{IF}\right]\in R^{n,K-1}$ . We cluster all *n* subjects by applying the *k*-means to the *n* rows of ${\hat{H}}^{IF}$ , assuming there are *K* clusters. Let $\hat{Y}={\left({\hat{Y}}_{1},{\hat{Y}}_{2},\ldots ,{\hat{Y}}_{n}\right)}^{\prime }$ be the predicted class labels.

To differentiate from the (orthodox) IF-PCA (which we call IF-PCA as follows), we call the aforementioned variant IF-PCA(X). See Table 1 in Section 2.7. The new variant was never proposed or studied before. It outperforms the (orthodox) IF-PCA in several data sets (e.g., see Section 3).

**TABLE 1:** Summary of all methods discussed in this section. This table clarifies the small differences between similar methods. Take the column IF-PCA(X) for example: “*W*” on row 2 means that the IF step of this method is applied to the normalized data matrix *W* defined in (2), and “*X*” on row 3 means the clustering step is applied to the un-normalized data matrix *X* (NA: not applicable).

	PCA	SpecGem	VAE	VAE(X)	IF-PCA	IF-PCA(X)	IF-VAE	IF-VAE(X)	Seurat	SC3
IF step	NA	NA	NA	NA	*W*	*W*	*W*	*W*	*X*	*X*
Clustering step	*X* or *W*	NA	*W*	*X*	*W*	*X*	*W*	*X*	*X*	*X*

### 2.5 IF-VAE and IF-VAE(X)

Near the end of Section 2.2, we mention that the classical PCA has two disadvantages: not exploiting sparsity in feature vectors and not accounting for possible non-linear relationships between the signal matrix and class labels. In Sections 2.3, 2.4, we have seen that the VAE aims to exploit non-linear relationships and IF-PCA aims to exploit sparsity. We may combine the VAE with the IF step of IF-PCA for simultaneously exploiting sparsity and non-linearity. To this end, we propose a new algorithm called IF-VAE.

The IF-VAE contains an IF step and a clustering step and runs as follows. Input: normalized data matrix $W=\left[w_{1},w_{2},\ldots ,w_{p}\right]={\left[W_{1},W_{2},\ldots ,W_{n}\right]}^{\prime }$ , number of classes *K*, and dimension of the latent space in the VAE (denoted by *d*). Output: predicted class label vector $\hat{Y}=\left({\hat{Y}}_{1},{\hat{Y}}_{2},\ldots ,{\hat{Y}}_{n}\right)$ .

• (*IF step*). We run the same IF step as in Section 2.4, and let $W^{IF}={\left[W_{1}^{IF},\ldots ,W_{n}^{IF}\right]}^{\prime }\in R^{n\times m}$ be the matrix consisting of the retained features only (same as in the IF step in IF-PCA, *m* is the number of retained features).
• (*Clustering step*). We apply the VAE with $W^{IF}\in R^{n\times m}$ and obtain an *n* × *d* matrix ${\hat{Z}}^{IF}$ , which can be viewed as an estimation of the low-dimensional representation of *W* ^*IF*^. We cluster the *n* samples into *K* clusters by applying the classical k-means to ${\hat{Z}}^{IF}$ assuming there are *K* classes. Let $\hat{Y}$ be the predicted label vector.

In the clustering step, we apply the VAE to the normalized data matrix *W*. Similarly, as in Section 2.4, if we apply the VAE to the un-normalized data matrix *X*, then we have a variant of IF-VAE, which we denote by IF-VAE(X). See Table 1 in Section 2.7.

### 2.6 Seurat and SC3

We now introduce Seurat and SC3, two recent algorithms that are especially popular for subject clustering with single-cell RNA-seq data. We discuss them separately.

Seurat was proposed in Satija et al. (2015). On a high level, Seurat is quite similar to IF-PCA, and we can view it as having only two main steps: a feature selection step and a clustering step. However, different from IF-PCA, Seurat uses a different feature selection step and a much more complicated clustering step (which combines several methods including PCA, k-nearest neighborhood algorithm, and modularity optimization). Seurat needs four tuning parameters: *m*, *N*, *k*_0_, *δ*, where *m* is the number of selected features in the feature selection step, and *N*, *k*_0_, *δ* are the clustering step, corresponding to the PCA part, the k-nearest neighborhood algorithm part, and the modularity optimization part, respectively.

A high-level sketch for Seurat is mentioned as follows (see Satija et al., 2015 for a more detailed description). Input: un-normalized *n* × *p* data matrix *X*, number of clusters *K*, and tuning parameters *m*, *N*, *k*_0_, *δ*. Output: predicted class label vectors $\hat{Y}={\left({\hat{Y}}_{1},{\hat{Y}}_{2},\ldots ,{\hat{Y}}_{n}\right)}^{\prime }$ .

• (IF step). We select the *m* features that are mostly variable. We obtain the *n* × *m* post-selection data matrix.
• (Clustering step). We normalize the post-selection data matrix and obtain the first *N* left singular vectors. For each pair of subjects, we compute how many neighbors (for each subject, we only count the *k*_0_ nearest neighbors) they share with each other and use the results to construct a shared nearest neighborhood (SNN) graph. We cluster the class labels by applying a modularity optimization algorithm to the SNN graph, where we need a resolution parameter *δ*.

An apparent limitation of Seurat is that it needs four tuning parameters. Following the recommendations by Hao et al. (2021), we may take (*N*, *k*_0_) = (50, 20), but it remains unclear how to select (*m*, *δ*).

SC3 was first presented by Kiselev et al. (2017). To be consistent with many other methods we discuss in this paper, we may view SC3 as containing two main steps: a gene-filtering step and a clustering step. Similar to Seurat, the clustering step of SC3 is much more complicated than that of IF-PCA, where the main idea is to apply PCA many times (each for a different number of leading singular vectors) and use the results to construct a matrix of consensus. We then cluster all subjects into *K* groups by applying the classical hierarchical clustering method to the consensus matrix. SC3 uses one tuning parameter *x*_0_ in the gene-filtering step and two tuning parameters, *d*_0_ and *k*_0_, in the clustering step, corresponding to the PCA part and the hierarchical clustering part, respectively.

A high-level sketch for SC3 is given as follows (see Kiselev et al., 2017 for a more detailed description). Input: un-normalized *n* × *p* data matrix *X*, true number of clusters *K*, and tuning parameters *x*_0_, *d*_0_, *k*_0_. Output: predicted class label vectors $\hat{Y}={\left({\hat{Y}}_{1},{\hat{Y}}_{2},\ldots ,{\hat{Y}}_{n}\right)}^{\prime }$ .

• (Gene-filtering step). Removes genes/transcripts that are either expressed (expression value is more than 2) in less than *x*_0_% of cells or expressed (expression value is more than 0) in at least (100 − *x*_0_)% of cells. This step may reduce a significant fraction of features, and we consider it to be more like a feature selection step than a preprocessing step.
• (Clustering step). First, we take a log-transformation of the post-filtering data matrix and construct an *n* × *n* matrix *M*, where *M*(*i*, *j*) is some kind of distance (e.g., Euclidean, Pearson, and Spearman) between subject *i* and *j*. Second, let $\hat{H}=\left[{\hat{\eta }}_{1},\ldots ,{\hat{\eta }}_{d}\right]$ , where ${\hat{\eta }}_{k}$ is the *k*th singular vector of *M* (or alternatively, of the normalized graph Laplacian matrix of *M*). Third, for *d* = 1, 2, … , *d*_0_, we cluster all *n* subjects to *K* classes by applying the k-means to the rows of the *n* × *d* sub-matrix of $\hat{H}$ consisting of the first *d* columns and use the results to build a consensus matrix using the Cluster-based Similarity Partitioning Algorithm (CSPA) (Strehl and Ghosh, 2002). Finally, we cluster the subjects by applying the classical hierarchical clustering to the consensus matrix with *k*_0_ levels of hierarchy.

Following the recommendation by Kiselev et al. (2017), we set (*x*_0_, *d*_0_) = (6, 15) and take *k*_0_ to be the true number of clusters *K*. Such a tuning parameter choice may work effectively in some cases, but for more general cases, we may (as partially mentioned in Kiselev et al. (2017)] need more complicated tuning.

In summary, on a high level, we can view both Seurat and SC3 as two-stage algorithms, which consist of a feature selection step and a clustering step, just as in IF-PCA. However, these methods use more complicated clustering steps, where the key is combining many different clustering results to reach a consensus; it is important to note that the SNN in Seurat can be viewed as a type of consensus matrix. Such additional steps taken in Seurat and SC3 may not only help reduce the clustering error rates but also make the algorithms conceptually more complex, computationally more expensive, and theoretically more difficult to analyze.

### 2.7 A brief summary of all the methods

We have introduced approximately 10 different methods, some of which (e.g., IF-PCA(X), IF-VAE, and IF-VAE(X)) have never never proposed before. Among these methods, the VAE is a popular UDL approach, Seurat and SC3 are especially popular in clustering with single-cell data, and IF-PCA is a conceptually simple method that has been shown to be effective in clustering with gene microarray data. It is important to note that some of the methods are conceptually similar to each other, with some small differences (though it is unclear how different their empirical performances are). For example, many of these methods are two-stage methods, containing an IF step and a clustering step. In the IF step, we usually use the normalized data matrix *W*. In the clustering step, we may use either *W* or the unnormalized data matrix *X*. To summarize all these methods and especially to clarify the small differences between similar methods, we have prepared a table given as follows; see Table 1 for details.

## 3 Results

Our study consists of two parts. In Section 3.1, we compare the IF-VAE with several other methods using 10 microarray data sets. In Section 3.2, we compare the IF-VAE with several other methods, including the popular approaches of Seurat and SC3, using eight single-cell data sets. In all these data sets, the class labels are given. However, we do not use the class labels in any of the clustering approaches; we only use them when we evaluate the error rates. The code for numerical results in this section can be found at https://github.com/ZhengTracyKe/IFPCA. The 10 microarray data sets can be downloaded at https://data.mendeley.com/datasets/cdsz2ddv3t, and the eight single-cell RNA-seq data sets can be downloaded at https://data.mendeley.com/drafts/nv2x6kf5rd.

### 3.1 Comparison of clustering approaches with 10 microarray data sets

Table 2 tabulates 10 gene microarray data sets (alphabetically) studied in Jin and Wang (2016). Here, data sets 1, 3, 4, 7, 8, and 9 were analyzed and cleaned in Dettling (2004) and data sets 2, 6, and 10 were analyzed and grouped into two classes in Yousefi et al. (2010), among which data set 10 was cleaned by Jin and Wang (2016) in the same way as Dettling (2004). Data set 5 is obtained from Gordon et al. (2002).

**TABLE 2:** Ten gene microarray data sets analyzed in Section 3.1 (*n*: number of subjects; *p*: number of genes; *K*: number of clusters).

#	Data name	Source	*K*	*n*	*p*
1	Brain	Pomeroy (02)	5	42	5,597
2	Breast cancer	Wang et al. (05)	2	276	22,215
3	Colon cancer	Alon et al. (99)	2	62	2,000
4	Leukemia	Golub et al. (99)	2	72	3,571
5	Lung cancer (1)	Gordon et al. (02)	2	181	12,533
6	Lung cancer (2)	Bhattacharjee et al. (01)	2	203	12,600
7	Lymphoma	Alizadeh et al. (00)	3	62	4,026
8	Prostate cancer	Singh et al. (02)	2	102	6,033
9	SRBCT	Kahn (01)	4	63	2,308
10	Su cancer	Su et al. (01)	2	174	7,909

First, we compare the IF-VAE approach introduced in Section 2.5 with four existing clustering methods: 1) the classical k-means; 2) Spectral-GEM (SpecGem) (Lee et al., 2010a), which is essentially classical PCA combined with a Laplacian normalization; 3) the orthodox IF-PCA (Jin and Wang, 2016), which adds a feature selection step prior to spectral clustering (see Section 2.4 for details); and 4) the VAE approach, which uses the VAE for dimension reduction and then runs k-means clustering (see Section 2.3 for details). Among these methods, SpecGem and VAE involve dimension reduction, and IF-PCA and IF-VAE use both dimension reduction and feature selection. For IF-PCA, VAE, and IF-VAE, we can implement the PCA step and the VAE step to either the original data matrix *X* or the normalized data matrix *W*. The version of IF-PCA associated with *X* is called IF-PCA(X), and the version associated with *W* is still called IF-PCA; similar rules apply to the VAE and IF-VAE. A total of eight different algorithms were obtained.

Table 3 shows the numbers of clustering errors (i.e., number of incorrectly clustered samples, subject to a permutation of *K* clusters) of these methods. The results of SpecGem and IF-PCA are copied from Jin and Wang (2016). We implemented k-means using the Python library sklearn, wrote the MATLAB code for IF-PCA(X), and wrote the Python code for the remaining four methods. The IF step of IF-VAE needs no tuning. In the VAE step of IF-VAE, we fix the latent dimension as *d* = 25 and use a traditional architecture in which both the encoder and decoder have one hidden layer; the encoder uses the ReLU activation, and the decoder uses the sigmoid activation; when training the encoder and decoder, we use a mini-batch stochastic gradient descent with 50 batches, 100 epochs, and a learning rate of 0.0005. The same neural network architecture and tuning parameters are applied to the VAE. We note that the outputs of these methods may have randomness due to the initialization in the k-means step or in the VAE step. For the VAE, IF-VAE, and IF-VAE(X), we repeat the algorithm 10 times and report the average clustering error. For k-means, we repeat it five times (because the results are more stable); for IF-PCA(X), we repeat it 20 times. We use the clustering errors to rank all eight methods for each data set; in the presence of ties, we assign ranks in a way such that the total rank sum is 36 (e.g., if two methods have the smallest error rate, we rank both of them as 1.5 and rank the second-best method as 3; other cases are similar). The average rank of a method is a metric of its overall performance across multiple data sets. In addition to ranks, we also compute regrets: for each data set, the *regret* of a method is defined to be *r* = (*e* − *e*_*min*_)/(*e*_*max*_ − *e*_*min*_), where *e* is the clustering error of this method and *e*_*max*_ and *e*_*min*_ are the maximum and minimum clustering error, respectively, among all the methods. The average regret also measures the overall performance of a method (the smaller, the better).

**TABLE 3:** Comparison of clustering errors of different methods with the 10 microarray data sets in Table 2. IF-PCA has the smallest average rank and average regret (boldface) and is regarded as the best on average.

Data set	k-means	SpecGem	IF-PCA	IF-PCA(X)	VAE	VAE(X)	IF-VAE	IF-VAE(X)
Brain	14	6	11	7	14	17	21	21
Breast cancer	121	121	112	91	105	130	120	118
Colon cancer	28	30	25	26	29	23	25	25
Leukemia	2	21	5	3	28	17	20	12
Lung cancer (1)	18	22	5	24	21	64	6	7
Lung cancer (2)	44	88	44	45	66	80	44	44
Lymphoma	1	14	1	18	23	22	16	10
Prostate cancer	43	43	39	44	41	45	42	41
SRBCT	28	32	28	24	33	26	30	23
Su cancer	83	85	58	57	62	60	57	57
Rank (mean)	4.3	6.1	**2.65**	3.9	5.7	5.8	4.3	3.25
Rank (SD)	2.07	2.20	1.18	2.33	2.20	2.35	1.90	1.74
Regret (mean)	0.43	0.69	**0.18**	0.26	0.60	0.65	0.46	0.31
Regret (SD)	0.35	0.33	0.22	0.32	0.33	0.39	0.36	0.33

There are several notable observations. First, somewhat surprisingly, the simple and tuning-free method, IF-PCA, has the best overall performance. It has the lowest average rank among all eight methods and achieves the smallest number of clustering errors in four out of 10 data sets. We recall that the key idea of IF-PCA is to add a tuning-free feature selection step prior to dimension reduction. The results in Table 2 confirm that this idea is highly effective with microarray data and hard to surpass by other methods. Second, the VAE (either on *W* or on *X*), which combines k-means with non-linear dimension reduction, significantly improves k-means on some “difficult” data sets, such as BreastCancer, ColonCancer, and SuCancer. However, for those “easy” datasets, such as Leukemia and Lymphoma, the VAE significantly underperforms compared to k-means. It suggests that the non-linear dimension reduction is useful mainly on “difficult” datasets. Third, the IF-VAE (either on *W* or on *X*) improves the VAE in the majority of data sets. In some datasets, such as LungCancer (1), the error rate of the IF-VAE is much lower than that of the VAE. This observation confirms that the IF step plays a key role in reducing the clustering errors. Jin and Wang (2016) made a similar observation by combining the IF step with linear dimension reduction by PCA. Our results suggest that the IF step continues to be effective when it is combined with non-linear dimension reduction by the VAE. Last, IF-VAE(X) achieves the lowest error rate in three out of 10 data sets, and it has the second lowest average rank among all eight methods. Compared with IF-PCA (the method with the lowest average rank), IF-VAE(X) has an advantage in three data sets (BreastCancer, SRBCT, and SuCancer) but has a similar or worse performance in the other data sets. These two methods share the same IF step; hence, the results imply that the non-linear dimension reduction by the VAE has an advantage over the linear dimension reduction by PCA only on “difficult” data sets.

**FIGURE 1:**
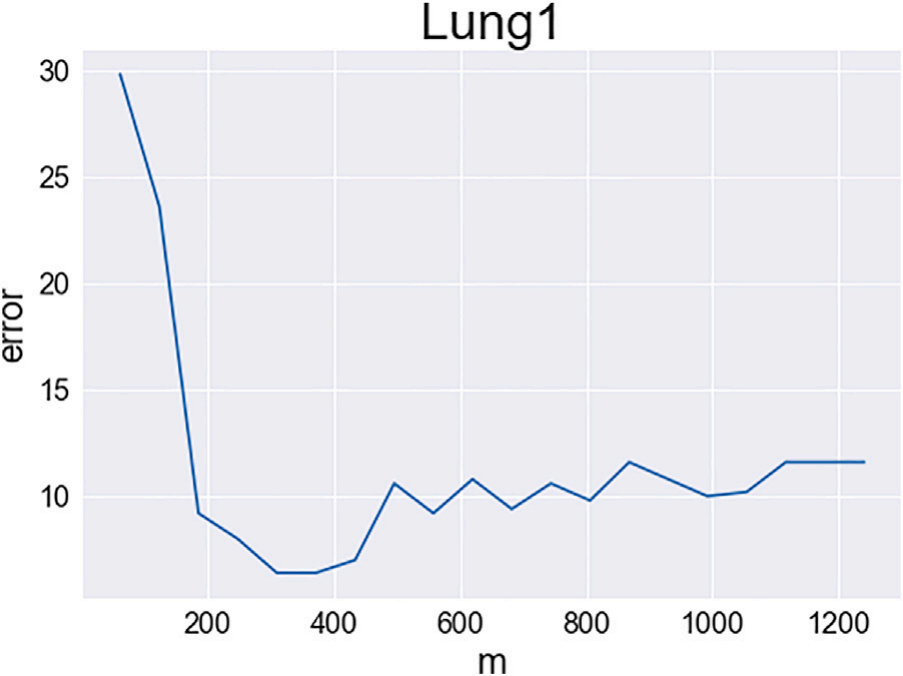
Clustering errors of IF-VAE(X) as a function of the number of selected features in the IF step (data set: LungCancer (1); *y*-axis: number of clustering errors; *x*-axis: number of selected features).

Next, we study IF-VAE(X) more carefully with the LungCancer (1) data set. We recall that the IF step ranks all the features using KS statistics and selects the number of features by a tuning-free procedure. We use the same feature ranking but manually change the number of retained features. For each *m*, we select the *m* top-ranked features, perform VAE on the unnormalized data matrix *X* restricted to these *m* features, and report the average number of clustering errors over five repetitions of the VAE. Figure 1 displays the number of clustering errors as a function of *m*. An interesting observation is that as *m* increases, the clustering error first decreases and then increases (for a good visualization; Figure 1 only shows the results for *m* between 1 and 0.1*p*; we also tried larger values of *m* and found that the number of clustering errors continued to increase; especially, the number of errors increased quickly when *m* > 4,000). A possible explanation is as follows: when *m* is too small, some influential features are missed, resulting in weak signals in the VAE step; when *m* is too large, too many non-influential features are selected, resulting in large noise in the VAE step. There is a sweet spot between 200 and 400, and the tuning-free procedure in the IF step selects *m* = 251. Figure 1 explains why the IF step benefits the subsequent VAE step. A similar phenomenon was discovered by Jin and Wang (2016), but it is for PCA instead of VAE.

Remark 1 (comparison with other clustering methods for microarray): Jin and Wang (2016) reported the clustering errors of several classical methods on these 10 microarray data sets. We only include k-means and SpecGem in Table 3 because k-means is the most widely used generic clustering method and SpecGem is specially designed for microarray data. Table 4 shows the clustering errors of other methods reported by Jin and Wang (2016), including k-means++ (a variant of k-means with a particular initialization) and hierarchical clustering. It suggests that these methods significantly underperform compared to IF-PCA.

**TABLE 4:** Clustering errors of k-means++ and hierarchical clustering with the 10 microarray data sets (the clustering errors of IF-PCA are listed for reference).

	Brain	Breast	Colon	Leuk	Lung 1	Lung 2	Lymph	Prostate	SRBCT	Su
k-means++	18	119	29	19	35	89	20	44	33	80
Hier	22	138	24	20	32	61	29	49	34	78
IF-PCA	11	112	25	5	5	44	1	39	28	58

### 3.2 Comparison of clustering approaches with eight single-cell RNA-seq datasets

Table 5 tabulates eight single-cell RNA-seq datasets. The data were downloaded from the Hemberg Group at the Sanger Institute (https://hemberg-lab.github.io/scRNA.seq.datasets). It contains scRNA-seq datasets from Human and Mouse. Among them, we selected eight datasets that have a sample size between 100 and 2,000 and can be successfully downloaded and pre-processed using the code provided by the Hemberg Group under the column ‘Scripts’. The datasets Camp1, Camp2, Darmanis, Li, and Patel come from Human, and the data sets Deng, Goolam, and Grun come from Mouse. Each data matrix contains the log-counts of the RNA-seq reads of different genes (features) in different cells (samples). The cell types are used as the true cluster labels to evaluate the performances of clustering methods. We first pre-processed all the data using the code provided by the Hemberg Group, then features (genes) with fractions of nonzero entries <5% are filtered out. The resulting dimensions for all datasets areshown in Table 5.

**TABLE 5:** Single-cell RNA-seq datasets investigated in this paper. (*n*: number of cells; *p*: number of genes; *K*: number of cell types).

#	Data set	*K*	*n*	*p*
1	Camp1	7	777	13,111
2	Camp2	6	734	11,233
3	Darmanis	9	466	13,400
4	Deng	6	268	16,347
5	Goolam	5	124	21,199
6	Grun	2	1,502	5,547
7	Li	9	561	25,369
8	Patel	5	430	5,948

We compare the IF-VAE with three other existing methods: 1) the orthodox IF-PCA (Jin and Wang, 2016), 2) Seurat (Satija et al., 2015), and 3) SC3 (Kiselev et al., 2017). The orthodox IF-PCA was proposed for subject clustering with microarray data. It is the first time this method has been applied to single-cell data. Seurat and SC3 are two popular methods clustering single-cell RNA-seq data (see Sections 2.6 for details). As discussed in Section 2.6, Seurat and SC3 implicitly use some feature selection ideas and some dimension reduction ideas, but they are much more complicated than IF-PCA and have several tuning parameters. Seurat has four tuning parameters, where *m* is the number of selected features, *N* is the number of principal components in use, *k*_0_ is the number of clusters in k-nearest neighbors, and *δ* is a ‘resolution’ parameter. We fix (*m*, *N*, *k*_0_) = (1,000, 50, 20) for all datasets (the values of (*N*, *k*_0_) are the default ones; the default value of *m* is 2,000, but we found that *m* = 1,000 gives the same results with the eight data sets and is faster to compute). We choose a separate value of *δ* for each dataset in a way such that the resulting number of clusters from a modularity optimization is exactly *K* [details can be found in Waltman and Van Eck (2013)]. Seurat is implemented by the R package Seurat (Hao et al., 2021). SC3 has three tuning parameters, where *x*_0_% is a threshold of the cell fraction used in the gene-filtering step, *d*_0_ is the number of eigenvectors in use, and *k*_0_ is the level of hierarchy in the hierarchical clustering step. We fix (*x*_0_, *d*_0_) = (10, 15) and set *k*_0_ as the number of true clusters in each dataset. SC3 is implemented using the R package SC3 (Kiselev et al., 2017). We observed that SC3 outputs an NA value on the Patel dataset because the gene filtering step removed all of the genes. To resolve this issue, we introduced a variant of SC3 by skipping the gene-filtering step. This variant is called SC3(NGF), where NGF stands for ‘no gene filtering.’ Seurat, SC3, and SC3(NGF) can only be applied to the unnormalized data matrix *X*. These methods also have randomness in the output, but the standard deviation of the clustering error is quite small; hence, we only run one repetition for each of them. The implementation of IF-PCA, IF-PCA(X), IF-VAE, and IF-VAE(X) is the same as shown in Section 3.1.

Table 6 contains the clustering accuracies (number of correctly clustered cells divided by the total number of cells) of different methods. For each dataset, we rank all six methods (excluding SC3) by their clustering accuracies (the higher the accuracy, the lower the rank). SC3 is excluded in rank calculation because it outputs NA on the Patel data set. Instead, we include SC3 (NGF), a version of SC3 that resolves this issue on Patel and has better performances in most other data sets; this gives more favor to SC3 in the comparison. For each data set, we also compute the regret of each method (the same as in Section 3.1). Similarly, we exclude SC3 but include SC3(NGF) in the regret calculation. Each method has a rank and a regret on each data set. The last four rows of Table 6 show the mean and standard deviation of the eight ranks of each method, as well as the mean and standard deviation of the eight regrets of each method.

**TABLE 6:** Comparison of the clustering accuracies with the eight single-cell RNA-seq datasets shown in Table 5. The result for SC3 on Patel is NA because all genes are removed in the gene-filtering step; for this reason, we exclude SC3 when calculating the rank and the regret. To resolve this issue, we also introduce a variant of SC3 by skipping the gene-filtering step. This variant is called SC3(NGF), where ‘NGF’ stands for no gene filtering. It performs better than the original SC3. It is important to note that IF-PCA(X) is regarded as the best on average: it has the smallest average regret (boldface) and average rank (boldface). It is also important to note that the standard deviation (SD) of its rank is only approximately 50% of that of SC3 (NGF).

Data set	Seurat	SC3	SC3(NGF)	IF-PCA	IF-PCA(X)	IF-VAE	IF-VAE(X)
Camp1	0.637	0.750	0.627	0.738	0.736	0.660	0.700
Camp2	0.661	0.713	0.759	0.601	0.656	0.393	0.491
Darmanis	0.682	0.826	0.867	0.635	0.747	0.406	0.617
Deng	0.530	0.590	0.754	0.791	0.588	0.607	0.687
Goolam	0.621	0.758	0.629	0.637	0.700	0.612	0.703
Grun	0.994	0.509	0.511	0.740	0.657	0.595	0.753
Li	0.934	0.938	0.980	0.889	0.968	0.848	0.853
Patel	0.898	NA	0.995	0.795	0.934	0.325	0.465
Rank (mean)	3.5	NA	**2.75**	3.0	**2.75**	5.38	3.63
Rank (SD)	1.7	NA	2.3	1.3	1.2	0.9	1.6
Regret (mean)	0.50	NA	0.37	0.40	**0.28**	0.90	0.53
Regret (SD)	0.4	NA	0.5	0.3	0.3	0.1	0.3

We make a few comments. First, if we measure the overall performance with eight data sets using the average rank, then IF-PCA(X) and SC3(NGF) are the best. If we use the average regret as the performance metric, then IF-PCA(X) is the best method. Second, a closer look at SC3(NGF) and IF-PCA(X) suggests that their performances have different patterns. SC3(NGF) is ranked 1 in some data sets (e.g., Camp2 and Darmanis) but has low ranks in some other data sets (e.g., Goolam and Grun). In contrast, IF-PCA(X) is ranked 2 in almost all data sets. Consequently, IF-PCA(X) has a smaller rank standard deviation, even though the two methods have the same average rank. One possible explanation is that SC3 is a complicated method with several tuning parameters. For some data sets, the current tuning parameters are appropriate, and so SC3 can achieve an extremely good accuracy; for some other data sets, the current tuning parameters are probably inappropriate, resulting in an unsatisfactory performance. In comparison, IF-PCA is a simple and tuning-free method and has more stable performances across multiple data sets. Third, IF-VAE(X) is uniformly better than the IF-VAE; hence, we recommend applying the IF-VAE to the unnormalized data matrix instead of the normalized one. Last, IF-VAE(X) significantly improves IF-PCA(X) on Deng and Grun. This suggests that the non-linear dimension reduction by the VAE is potentially useful with these two data sets. In the other data sets, IF-VAE(X) either underperforms compared to IF-PCA(X) or performs similarly.

In terms of computational costs, Seurat is the fastest, followed by IF-PCA. VAE and SC3 are more time-consuming, where the main cost of VAE arises from training the neural network, and the main cost of SC3 arises from computing the *n* × *n* similarity matrix among subjects. For a direct comparison, we report the running time of different methods with the Camp1 data set (*n* = 777 and *p* = 13,111). IF-PCA is implemented in MATLAB and takes approximately 1.7 min. The VAE and IF-VAE are implemented in Python, where the VAE steps are conducted using the Python library keras. The running time of the VAE is 2.7 min, and the running time of the IF-VAE is 1.4 min. SC3 is implemented via the package SC3 of Bioconductor in R, and it takes 3 min. Seurat is implemented using the R package Seurat and takes only 6 s.

Remark 2 (using ARI as the performance metric): The Adjusted Rand Index (ARI) is another commonly used metric for clustering performance. As shown in Table 7, we report the ARI of different methods and recalculate the ranks and regrets. The results are quite similar to those in Table 6.

**TABLE 7:** Values of the Adjusted Rand Index (ARI) for the same data sets and methods as shown in Table 6. Similarly, the average rank and regret of SC3 are denoted as NA, for it generated NA with the Patel data set.

Data set	Seurat	SC3	SC3(NGF)	IF-PCA	IF-PCA(X)	IF-VAE	IF-VAE(X)
Camp1	0.534	0.768	0.526	0.628	0.627	0.606	0.615
Camp2	0.443	0.577	0.502	0.410	0.493	0.162	0.304
Darmanis	0.480	0.682	0.784	0.489	0.650	0.219	0.525
Deng	0.442	0.646	0.669	0.771	0.477	0.487	0.555
Goolam	0.543	0.687	0.544	0.356	0.562	0.410	0.534
Grun	0.969	−0.066	−0.060	0.135	0.102	0.023	0.137
Li	0.904	0.951	0.968	0.797	0.940	0.798	0.792
Patel	0.790	NA	0.989	0.598	0.850	0.173	0.235
Rank (mean)	3.62	NA	**2.50**	3.50	**2.50**	5.00	3.88
Rank (SD)	1.60	NA	2.20	1.77	1.31	0.93	1.36
Regret (mean)	0.42	NA	0.30	0.51	**0.29**	0.84	0.59
Regret (SD)	0.37	NA	0.44	0.40	0.37	0.27	0.33

The bold values indicate the smallest value in that row.

Remark 3 (comparison with RaceID): In addition to Seurat and SC3, there are many other clustering methods for single-cell data (e.g., see Yu et al. (2022) for a survey). RaceID (Grün et al., 2015) is a recent method. It runs an initial clustering, followed by an outlier identification; and the outlier identification is based on a background model of combined technical and biological variability in single-cell RNA-seq measurements. We now compare IF-PCA(X) and IF-VAE(X) with RaceID (we used the R package RaceID and set all tuning parameters to be at default values in this package). The results are shown in Table 8. We observe that IF-PCA(X) and IF-VAE(X) outperform RaceID with most data sets. One possible reason is that the outlier identification step in RaceID is probably more suitable for applications with a large number of cells (e.g., tens of thousands of cells).

**TABLE 8:** Comparison of the clustering accuracies of IF-PCA(X), IF-VAE(X), and RaceID.

	Camp1	Camp2	Darmanis	Deng	Goolam	Grun	Li	Patel
IF-PCA(X)	0.736	0.656	0.747	0.588	0.700	0.657	0.968	0.934
IF-VAE(X)	0.700	0.491	0.617	0.687	0.703	0.753	0.853	0.465
RaceID	0.645	0.425	0.290	0.630	0.443	0.583	0.624	0.542

Remark 4 (combining the IF step with Seurat and SC3): We investigate whether the IF step of IF-PCA can be used to conduct feature selection for other clustering methods. To this end, we introduce IF-Seurat and IF-SC3(NGF), in which Seurat and SC3(NGF) are applied, respectively, to the post-selection unnormalized data matrix from the IF step of IF-PCA. Table 9 compares these two methods with their original versions. For Seurat, the IF step improves the clustering accuracies on Camp1, Darmanis, and Patel; yields similar performances on Deng, Goolam Grun, and Li; and deteriorates the performances significantly on Camp2. For SC3, the IF step sometimes yields a significant improvement (e.g., Camp1) and sometimes a significant deterioration (e.g., Deng). It is an interesting theoretical question when the current IF step is suitable to combine with clustering methods other than PCA.

**TABLE 9:** Combinations of IF-Seurat with Seurat and IF-SC3(NGF) with SC3(NGF).

	Camp1	Camp2	Darmanis	Deng	Goolam	Grun	Li	Patel
Seurat	0.637	0.661	0.682	0.530	0.621	0.994	0.934	0.898
IF-Seurat	0.647	0.485	0.779	0.526	0.597	0.986	0.879	0.937
SC3(NGF)	0.627	0.759	0.867	0.754	0.629	0.511	0.980	0.995
IF-SC3(NGF)	0.724	0.702	0.796	0.489	0.637	0.550	0.998	0.981

## 4 Phase transition for PCA and IF-PCA

Compared with VAE, Seurat, and SC3, an advantage of IF-PCA is that it is conceptually much simpler and thus comparably easier to analyze. In this section, we present some theoretical results and show that IF-PCA is optimal in a rare/weak signal setting.

We are interested in several intertwined questions.

• When the IF step of the IF-PCA is really necessary. As IF-PCA reduces to classical PCA when we omit the IF step, an equivalent question is when IF-PCA really has an advantage over PCA.
• When IF-PCA is optimal in a minimax decision framework.

To facilitate the analysis, we consider a high-dimensional clustering setting where *K* = 2, so we only have two classes. We assume the two classes are equally likely so that the class labels satisfy

$$Y_{i}\overset{iid}{∼}2Bernoulli\left(1/2\right)-1,\;1\leq i\leq n;$$ (10)

extension to the case where we replace the Bernoulli parameter 1/2 by a *δ* ∈ (0, 1), which is comparably straightforward. We also assume that the *p*-dimensional data vectors *X*_*i*_’s are standardized so that for a contrast mean vector *μ* ∈ *R* ^*p*^ (*I*_*p*_ standards for the *p* × *p* identity matrix),

$$X_{i}=Y_{i}\mu +Z_{i},\;Z_{i}\overset{iid}{∼}N\left(0,I_{p}\right),\;1\leq i\leq n.$$ (11)

As mentioned before, we write $Y={\left(Y_{1},Y_{2},\ldots ,Y_{n}\right)}^{\prime }$ , $X={\left[X_{1},X_{2},\ldots ,X_{n}\right]}^{\prime }=\left[x_{1},x_{2},\ldots ,x_{p}\right]$ . It follows

$$X=Y\mu^{\prime }+Z,\;\text{where similarly}\;\;Z={\left[Z_{1},Z_{2},\ldots ,Z_{n}\right]}^{\prime }=\left[z_{1},z_{2},\ldots ,z_{p}\right].$$

For any 1 ≤ *j* ≤ *p*, we call feature *j* an “influential feature” or “useless feature” if *μ*(*j*) ≠ 0 and a “noise” or “useless feature” otherwise. We adopt a rare/weak model setting where (*ν*_*a*_ stands for point mass at *a*)

$$\mu \left(j\right)\overset{iid}{∼}\left(1-\epsilon_{p}\right)\nu_{0}+\left(\epsilon_{p}/2\right)\nu_{\tau_{p}}+\left(\epsilon_{p}/2\right)\nu_{-\tau_{p}}.$$ (12)

For fixed parameters 0 < *θ*, *β*, *α* < 1,

$$n=n_{p}=p^{\theta },\;\epsilon_{p}=p^{-\beta },\;\tau_{p}=p^{-\alpha }.$$ (13)

From time to time, we drop the subscript of *n*_*p*_ and write *n* = *n*_*p*_. For later use, let

$$s_{p}=p\epsilon_{p}\;\text{and}\;S_{p}\left(\mu \right)=\left\{1\leq j\leq p:\mu \left(j\right)\neq 0\right\}\;\;\text{be the support of}\;\;\mu .$$ (14)

It is seen that |*S*_*p*_(*μ*)|∼Bernoulli (*p*, *ϵ*_*p*_) and |*S*_*p*_(*μ*)|/*s*_*p*_ ∼ 1. Models (10)–(13) model a scenario where 1 ≪ *n* ≪ *p* and

• (Signals are sparse/rare). The fraction of the influential feature is *p* ^−*β*^, which → 0 rapidly as *p* → *∞*.
• (Signals are individually weak). The signal strength of each influential feature may be much smaller than *n* ^−1/4^, and the signals are individually weak; it is non-trivial to separate the useful features from the useless ones.
• (No free lunch). Summing over *X* either across rows (samples) or across columns (feature) would not provide any useful information for clustering decisions.

The model is frequently used if we want to study the fundamental limits and phase transition associated with a high-dimensional statistical decision problem (e.g., classification, clustering, and global testing). Despite the seeming simplicity, the RW model is actually very delicate to study, for it models a setting where the signals (i.e., useful features) are both rare and weak. See Donoho and Jin (2004); Jager and Wellner (2007); Hall and Jin (2010); Xie et al. (2011); Donoho and Jin (2015); Verzelen and Arias-Castro (2017) for example.

Compared with the model in Jin and Wang (2016) (which only considers one-sided signals, where all non-zero *μ*(*j*) are positive), our model allows the two-sided signal and so is different. In particular, in our model, summing over *X* either across rows or columns would not provide any useful information for clustering decisions. As a result, the phase transition we derive as follows is different from that in Jin and Wang( 2016).

We consider a clustering procedure and let $\hat{Y}\in R^{n}$ be the predicted class label vector. It is important to note that for any 1 ≤ *i* ≤ *n*, both *Y*_*i*_ (true class label) and ${\hat{Y}}_{i}$ take values from { − 1, 1}. Let Π be the set of all possible permutations on { − 1, 1}. We measure the performance of $\hat{Y}$ by the Hamming error rate:

$${Hamm}_{p}\left(\hat{Y},Y\right)={Hamm}_{p}\left(\hat{Y},Y;\beta ,\theta \right)=n^{-1}\inf_{\pi_{0}\in \Pi }\left\{\overset{n}{\underset{i=1}{\sum }}P\left({\hat{Y}}_{i}\neq \pi_{0}Y_{i}\right)\right\},$$ (15)

where the probability measure is with respect to the randomness of (*μ*, *Y*, *Z*).

### 4.1 A slightly simplified version of PCA and IF-PCA

To facilitate analysis for Models (10)–(13), we consider a slightly more idealized version of PCA and IF-PCA, where the main changes are 1) we skip the normalization step (as we assume the model is for data that are already normalized); 2) we replace feature selection by Kolmogorov–Smirnov statistics in IF-PCA by feature selection by the *χ* ^2^ statistics; 3) we remove Efron’s correction in IF-PCA (Efron’s correction is especially useful for analyzing gene microarray data, but is not necessary for the current model); and 4) we skip the HCT choice (the study on HCT is quite relevant for our model, but technically it is very long, so we skip it). It is also important to note that the rank of the signal matrix *Yμ*′ is 1 in Models (10)–(13), so in both PCA and the clustering step of IF-PCA, we should apply k-means clustering to the first singular vector of *X* only. Despite these simplifications, the essences of original PCA and IF-PCA are retained. A more detailed description of the (simplified) PCA and IF-PCA is as follows.

In detail, to use PCA for Models (10)–(13), we run the following.

• We obtain the first singular vector of *X* and denote it by *ξ* (this is simpler than $\hat{\xi }$ ; we are misusing the notation a little bit here).
• We cluster by letting ${\hat{Y}}_{i}=\mathrm{sgn}\left(\xi_{i}\right)$ , 1 ≤ *i* ≤ *n*.

To be differentiable from PCA in Section 2.2, we may call the approach *the slightly simplified PCA*.

In addition, to use IF-PCA for Models (1)–(4), we introduce the normalized *χ* ^2^-testing scores for feature *j* by

$$\psi_{j}=\left(\|x_{j}{\|}^{2}-n\right)/\sqrt{2n}.$$ (16)

By elementary statistics,

$$\psi_{j}∼\left\{\begin{array}{cc} N\left(\sqrt{\left(n/2\right)}\tau_{p}^{2},1\right),\; & \;\text{if feature}\;\;j\;\;\text{is useful},\\ N\left(0,1\right),\; & \;\text{otherwise}. \end{array}\right.$$

Fix a threshold

$$t_{p}^{*}=\sqrt{2\log\left(p\right)}.$$

The IF-PCA runs as follows.

• (IF step). We select feature *j* if and only if $\psi_{j}\geq t_{p}^{*}$ .
• (Clustering step). Let

$$\hat{S}=\left\{1\leq j\leq p:\psi_{j}\geq t_{p}^{*}\right\},$$

and let $X_{\hat{S}}$ be the post-selection data matrix (which is a sub-matrix of *X* consisting of columns in $\hat{S}$ ). Let $\xi^{*}\in R^{n}$ be the first singular vector of ${\hat{X}}_{S}$ . We cluster by letting

$${\hat{Y}}_{i}=\mathrm{sgn}\left(\xi_{i}^{*}\right),\;1\leq i\leq p.$$

Similarly, to differentiate from the IF-PCA in Section 2.4, we call this the slightly simplified IF-PCA.

### 4.2 The computational lower bound

We first discuss the computational lower bound (CLB). The notion of CLB is an extension of the classical information lower bound (LB) (e.g., the Cramer–Rao lower bound), and in comparison,

• Classical information LB usually claims a certain goal is not achievable for any method (which includes methods that are computationally NP hard).
• CLB usually claims a certain goal is not achievable for any method with *a polynomial computational time.*

From a computational perspective, we highly prefer to have algorithms with a polynomial computation time. Therefore, compared with classical information LB, CLB is practically more relevant.

Let *s*_*p*_ = *pϵ*_*p*_. It is important to note that in our model, the number of signals is Bernoulli (*p*, *ϵ*_*p*_), which concentrates at *s*_*p*_. We recall that in our calibrations, *n* = *p* ^*θ*^ and *s*_*p*_ = *p* ^1−*β*^, and the strength of individual signals is *τ*_*p*_. We introduce the critical signal strength by

$$\tau_{p}^{*}=\left\{\begin{array}{cc} {\left[p/\left(ns_{p}^{2}\right)\right]}^{1/4},\; & \;\text{if}\;\beta <1/2\left(\text{so}\;\;s_{p}\gg \sqrt{p}\right),\\ n^{-1/4},\; & \;\text{if}\;1/2<\beta <\left(1-\theta /2\right)\left(\text{so}\sqrt{n}\ll s_{p}\ll \sqrt{p}\right).\\ s_{p}^{-1/2},\; & \;\text{if}\;\left(1-\theta /2\right)<\beta <1\left(\text{so}\;\;1\ll s_{p}\ll \sqrt{n}\right). \end{array}\right.$$

We have the following theorem.

Theorem 4.1 [Computational lower bound)]. We fix (*θ*, *β*) ∈ (0,1)^2^ and consider the clustering problem for Models (10)–(13). *As* *p* → *∞* *, if* $\tau_{p}/\tau_{p}^{*}\to 0$ , then for any clustering procedure $\hat{Y}$ with a polynomial computational time, ${Hamm}_{p}\left(\hat{Y},Y\right)\geq \left(1/2+o\left(1\right)\right)$ *.*

In other words, any “computable clustering procedure” (meaning those with a polynomial computational time) fails in this case, where the error rate is approximately the same as that of random guess. The proof of Theorem 4.1 is long but is similar to that of [Jin et al. (2017), Theorem 1.1], so we omit it.

Next, we study the performance of classical PCA and IF-PCA. However, before we perform that, we present a lemma on classical PCA in Section 4.3. We state the lemma in a setting that is more general than Models (10)–(13), but we will come back to Models (10)–(13) in Section 4.4.

### 4.3 A useful lemma on classical PCA

Suppose we have a data matrix $X\in R^{N,m}$ in the form of

$$X=Y\mu^{\prime }+Z,\;Y\in R^{N},\mu \in R^{m}.$$ (17)

In such a setting, we investigate when the PCA approach in Section 4.1 is successful. We recall that *ξ* is the first singular vector of *X*. By basic algebra, it is the first eigenvector of the *N* × *N* matrix *XX*′, or equivalently, the first eigenvector of *XX*′ − *mI*_*N*_. We write

$$\begin{array}{ll} XX^{\prime }-mI_{N} & =\|\mu {\|}^{2}YY^{\prime }+\left(ZZ^{\prime }-mI_{N}\right)+\left(Y\mu^{\prime }Z^{\prime }+Z\mu Y^{\prime }\right)\\ & =\|\mu {\|}^{2}\cdot YY^{\prime }+\left(ZZ^{\prime }-mI_{N}\right)+\text{secondary term}. \end{array}$$

In order for the PCA approach to be successful, the spectral norm of ‖*μ*‖^2^ *YY*′ should be much larger than that of (*ZZ*′ − *mI*_*N*_). It is important to note that ‖*μ*‖^2^ *YY*′ is a rank-1 matrix, where the spectral norm is *N*‖*μ*‖^2^. In addition, by random matrix theory (Vershynin, 2012), the spectral norm of (*ZZ*′ − *mI*_*N*_) concentrates at ${\left(\sqrt{N}+\sqrt{m}\right)}^{2}-m=N+2\sqrt{Nm}$ . Therefore, the main condition we need for the PCA approach to be successful is

$$N\|\mu {\|}^{2}/\left(N+2\sqrt{Nm}\right)\to \infty .$$ (18)

We have the following lemma.

Lemma 4.1 Here, we consider Model (8) where condition (9) holds and that ‖*μ*‖^2^ ≫ log(*N* + *m*). Let *ξ* be the first left singular vector of *X*. When min{*N*, *m*} → *∞*, with probability 1 − *o*(*m* ^−3^),

$$\min\left\{\|\sqrt{N}\xi +Y{\|}_{\infty },\|\sqrt{N}\xi -Y{\|}_{\infty }\right\}=o\left(1\right).$$

Lemma 4.1 is proved in the Supplementary Material. This result connects to the recent interests of studying entry-wise large-deviation bounds of eigenvectors (Abbe et al., 2020; Fan et al., 2022). Our proof is based on a form of Taylor expansion of eigenvectors. Please see the Supplementary Material for details.

By Lemma 4.1, there is an error vector *r* with ‖*r*‖_*∞*_ = *o* (1) such that

$$\sqrt{N}\xi =\pm Y+r;\;\text{recall that }Y_{i}\in \left\{-1,1\right\}.$$

Therefore, if we let ${\hat{Y}}_{i}=\mathrm{sgn}\left(\xi_{i}\right)$ as in the PCA approach in Section 4.1, then except for a small probability,

$$\hat{Y}=\pm Y.$$

This says that the PCA approach can fully recover the true class labels.

### 4.4 Achievability of classical PCA and IF-PCA

We now come back to Models (10)–(13) and study the behavior of classical PCA and IF-PCA in our setting. The computational limits of clustering have received extensive interest [e.g., (Luo and Zhang, 2022)]. By the CLB (Jin et al., 2017), successful clustering by a computable algorithm is impossible when $\frac{\tau_{p}}{\tau_{p}^{*}}\to 0$ , so the interesting parameter range for PCA and IF-PCA is when

$$\tau_{p}/\tau_{p}^{*}\to \infty .$$

We first discuss when feature selection by *χ* ^2^-test is feasible. As mentioned before, let

$$\psi_{j}={\left(2n\right)}^{-1/2}\left(\|x_{j}{\|}^{2}-n\right),$$

be the feature-wise *χ* ^2^-testing scores and recall that approximately,

$$\psi_{j}∼\left\{\begin{array}{cc} N\left(\sqrt{\left(n/2\right)}\tau_{p}^{2},1\right),\; & \;\text{if feature}\;j\;\text{is useful},\\ N\left(0,1\right),\; & \;\text{otherwise}. \end{array}\right.$$

We can view $\sqrt{\left(n/2\right)}\tau_{p}^{2}$ as the signal-to-noise ratio (SNR) for the *χ* ^2^-test for a useful feature. We have two cases.

• (Less sparse case of *β* < 1/2). In this case, the number of useful features *s*_*p*_ is much larger than $\sqrt{p}$ and $\tau_{p}^{*}\ll n^{-1/4}$ , and the SNR of *ψ*_*j*_ for a useful feature *j* may be much smaller than 1, even though $\tau_{p}/\tau_{p}^{*}\to \infty$ . In such a case, feature selection by the *χ* ^2^-test is not useful. Consequently, except for a negligible probability, the IF step of IF-PCA selects all features, so IF-PCA reduces to PCA.
• (More sparse case of *β* > 1/2). In this case, the number of useful features *s*_*p*_ is much smaller than $\sqrt{p}$ and $\tau_{p}^{*}\geq n^{-1/4}$ . If $\tau_{p}/\tau_{p}^{*}\to \infty$ , then the SNR of *ψ*_*j*_ → *∞* if *j* is a useful feature. In such a case, feature selection maybe successful and IF-PCA is significantly different from PCA.

Consider the first case and suppose we apply the PCA approach in Section 4.1 directly to matrix *X*. Applying Lemma 4.1 with (*N*, *m*) = (*n*, *p*) and noting that in this setting,

$$n\|\mu {\|}^{2}∼ns_{p}\tau_{p}^{2},N+2\sqrt{Nm}=p+2\sqrt{np}∼2\sqrt{np}\;\;\left(\text{since}\;\;n\ll p\right),$$

the PCA approach is successful if

$$ns_{p}\tau_{p}^{2}/\sqrt{np}\to \infty .$$

Comparing this with the definition of $\tau_{p}^{*}$ , this is equivalent to

$$\tau_{p}/\tau_{p}^{*}\to \infty ,\;\text{as}\;\;0<\beta <1/2\;\;\text{in the current case}.$$

We have the following theorem.

Theorem 4.2 (Possibility *Region* for PCA). We fix (*θ*, *β*) ∈ (0,1)^2^ and consider the clustering problem for Models (1)–(4). Let ${\hat{Y}}^{\mathrm{pca}}$ be the predicted class label vector by the PCA algorithm in Section 4.1. As *p* → *∞*, if

$$0<\beta <1/2\;\left(\mathrm{so}\;s_{p}/\sqrt{p}\to \infty \right)\;\text{and}\;\frac{\tau_{p}}{\tau_{p}^{*}}\to \infty ,$$ (19)

then ${Hamm}_{p}\left({\hat{Y}}^{\mathrm{pca}},Y\right)\to 0$ *.*

Consider the second case, where we may have successful feature selection, so it is desirable to use IF-PCA. We assume

$$\tau_{p}/\tau_{p}^{*}\geq {\left(4\log\left(p\right)\right)}^{1/4},$$ (20)

which is slightly stronger than that of $\tau_{p}^{*}/\tau_{p}\to \infty$ . By the definition of $\tau_{p}^{*}$ , we have that in the current case (where 1/2 < *β* < 1)

$$\tau_{p}^{*}\geq n^{-1/4}.$$ (21)

We recall that *S*(*μ*) is the true support of *μ* and

$$\hat{S}=\left\{1\leq j\leq p:\psi_{j}\geq \sqrt{2\log\left(p\right)}\right\},$$

is the set of selected features in the IF step of IF-PCA. We recall that

$$\psi_{j}∼\left\{\begin{array}{cc} N\left(\sqrt{\left(n/2\right)}\tau_{p}^{2},1\right),\; & \;\text{if feature}\;\;j\;\;\text{is useful},\\ N\left(0,1\right),\; & \;\text{otherwise}. \end{array}\right.$$

By (20, 21), for any useful feature *j*, the SNR is

$$∼\sqrt{\left(n/2\right)}\tau_{p}^{2}\geq \sqrt{\left(n/2\right)}\sqrt{4\log\left(p\right)}n^{-1/2}=\sqrt{2\log\left(p\right)}.$$

By elementary statistics, we have that approximately,

$$P\left(\hat{S}\neq S\right)=o\left(1\right),\;\text{where for short}\;\;S=S\left(\mu \right);\text{same below}.$$

Therefore, except for a negligible probability,

$$X_{\hat{S}}=X_{S}=Y\mu_{S}^{\prime }+Z_{S},$$

where similar as before, *μ*_*S*_ is the sub-vector of *μ* with all entries restricted to *S*, and *X*_*S*_ and *Z*_*S*_ are the sub-matrices of *X* and *Z* respectively, with columns restricted to *S*. Therefore, in the clustering step of IF-PCA, we are, in effect, applying the PCA approach of Section 4.1 to *X*_*S*_, where we recall |*S*|/*s*_*p*_ ≈ 1. Applying Lemma 4.1 with (*N*, *m*) = (*n*, |*S*|) and noting that

$$n\|\mu_{S}{\|}^{2}∼ns_{p}\tau_{p}^{2},\;N+2\sqrt{Nm}=n+2\sqrt{n|S|}∼n+2\sqrt{ns_{p}},$$

it follows that in order for the clustering step of IF-PCA to be successful, we need

$$ns_{p}\tau_{p}^{2}/\left(n+2\sqrt{ns_{p}}\right)\to \infty ,\;\left(\text{note that when}\;\;s_{p}\ll n,\;\text{this is equivalent to}\;s_{p}\tau_{p}^{2}\to \infty \right).$$ (22)

Combining this with (22) and recalling that in the current case, $s_{p}\ll \sqrt{p}$ , IF-PCA is successful when

$$\left\{\begin{array}{cc} \tau_{p}^{2}\geq 2\sqrt{\log\left(p\right)/n},\; & \;\text{if}\;\;\sqrt{n}\ll s_{p}\ll \sqrt{p},\\ s_{p}\tau_{p}^{2}\to \infty ,\; & \;\text{if}\;\;s_{p}\ll \sqrt{n}. \end{array}\right.$$ (23)

Comparing this with the definition of $\tau_{p}^{*}$ , (23) holds if we assume

$$\tau_{p}/\left(\sqrt{\log\left(p\right)}\tau_{p}^{*}\right)\to \infty ,$$

which is slightly stronger than that of $\tau_{p}/\tau_{p}^{*}\to \infty$ . We have the following theorem.

Theorem 4.3 (Possibility *Region* for IF-PCA). We fix (*θ*, *β*) ∈ (0,1)^2^ and consider the clustering problem for Models (10)–(13). Let ${\hat{Y}}^{\mathrm{ifpca}}$ be the predicted class label vector by the PCA algorithm in Section 4. As *p* → *∞*, if

$$1/2<\beta <1\;\left(\text{so}\;\;s_{p}/\sqrt{p}\to 0\right)\;\text{and}\;\frac{\tau_{p}}{\sqrt{\log\left(p\right)}\tau_{p}^{*}}\to \infty ,$$ (24)

then, in the IF step of IF-PCA,

$$P\left(\hat{S}\neq S\left(\mu \right)\right)=o\left(1\right).$$

Moreover, ${Hamm}_{p}\left({\hat{Y}}^{\mathrm{ifpca}},Y\right)\to 0$ *.*

### 4.5 Phase transition

We recall that *s*_*p*_ = *pϵ*_*p*_ and that in Models (10)–(13),

$$n=n_{p}=p^{\theta },\;\epsilon_{p}=p^{-\beta },\;\tau_{p}=p^{-\alpha }.$$

It follows

$$\tau_{p}^{*}=p^{-\alpha^{*}\left(\beta ,\theta \right)},\;\text{where}\;\alpha^{*}\left(\beta ,\theta \right)=\left\{\begin{array}{cc} \left(1+\theta -2\beta \right)/4,\; & \;\text{if}\;\;0<\beta <1/2,\\ \theta /4,\; & \;\text{if}\;\;1/2<\beta <1-\theta /2,\\ \left(1-\beta \right)/2,\; & \;\text{if}\;\;\left(1-\theta /2\right)<\beta <1. \end{array}\right.$$

We fix 0 < *θ* < 1 and consider the two-dimensional space where the two axes are *β* and *α*, respectively. Combining Theorems 4.2, 4.3, the curve *α* = α*(*β*, *θ*) partitions the region {(*α*, *β*): 0 < *β* < 1, *α* > 0} into two regions.

**FIGURE 2:**
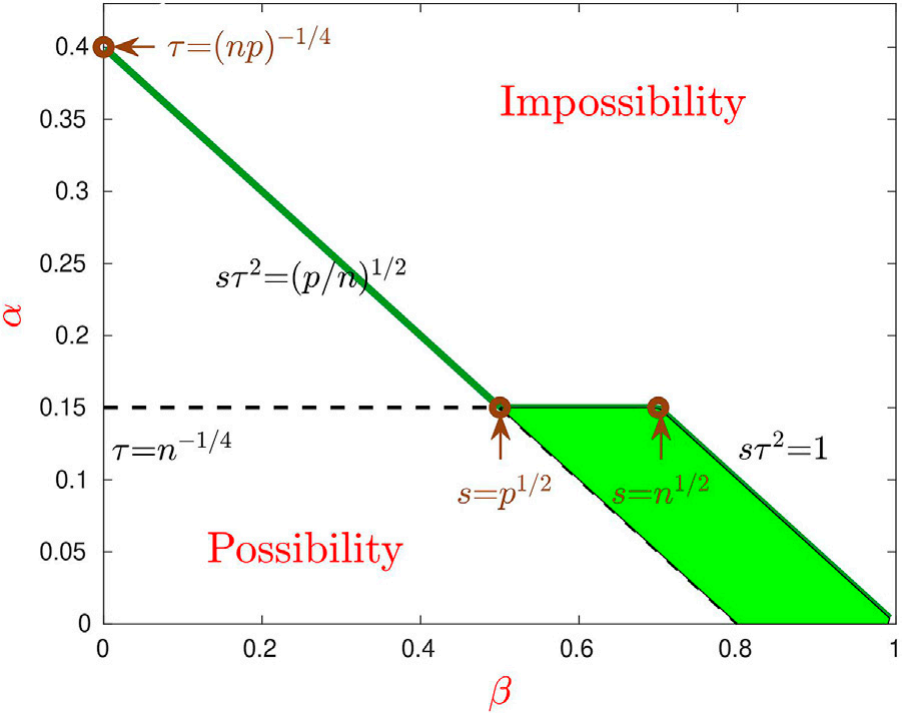
Phase transition for PCA and IF-PCA (*θ* = 0.6). The (three-segment) solid green line is *α* = α*(*β*, *θ*), which separates the whole region into the Region of Impossibility (top) and Region of Possibility (bottom). In the part of Region of Possibility (*β* < 1/2), feature selection is infeasible, PCA is optimal, and IF-PCA reduces to PCA with an appropriate threshold. In the right part (*β* > 1/2), it is desirable to conduct feature selection, and IF-PCA is optimal. However, PCA is non-optimal for parameters in the shaded green region.

• Region of *Im*possibility {(*α*, *β*): *α* > α*(*β*, *θ*), 0 < *β* < 1}. In this region, the Hamming clustering error rate of any method with polynomial computation time is bounded away from 0.
• Region of *Poss*ibility {(*α*, *β*): *α* < α*(*β*, *θ*), 0 < *β* < 1}. The region further partitions into two parts: *β* < 1/2 (left) and *β* > 1/2 (right).
  • The left is the *less sparse case* where the number of useful features $s_{p}\gg \sqrt{p}$ . For any fixed (*α*, *β*) in this region, the Hamming error rate of PCA is *o*(1), so PCA achieves the optimal phase transition. In addition, in this case, the signals are too weak individually and feature selection is infeasible. Therefore, in the IF step, the best we can do is to select all features, so IF-PCA reduces to PCA.
  • The right is the more sparse case, where the number of useful features $s_{p}\ll \sqrt{p}$ . For any fixed (*α*, *β*) in this region, the Hamming error rate of IF-PCA is *o*(1), so IF-PCA achieves the optimal phase transition. In addition, in this case, the signals are strong enough individually and feature selection is desirable. Therefore, IF-PCA and PCA are significantly different.
  • In particular, for any fixed parameters in the region {1/2 < *β* < 1, (1 − *θ* − 2*β*) < *α* < (1 − *β*)/2} (shaded green region of Figure 2), the Hamming clustering error rate of IF-PCA is *o* (1), but that of PCA is bounded away from 0. Therefore, PCA is non-optimal in this particular region.

See Figure 2 for details.

## 5 Discussion

IF-PCA is a simple and tuning-free approach to unsupervised clustering of high-dimensional data. The main idea of IF-PCA is a proper combination of feature selection and dimension reduction by PCA. In this study, we make several contributions. First, we extend IF-PCA to IF-VAE, by replacing PCA with the VAE, a popular UDL algorithm. Second, we study the theoretical properties of IF-PCA in a simple clustering model and derive the phase transitions. Our results reveal how the feature sparsity and the feature strength affect the performance of IF-PCA and explain why IF-PCA can significantly improve the classical PCA. Third, we investigate the performances of IF-PCA and IF-VAE on two applications, the subject clustering with gene microarray data and the cell clustering with single-cell RNA-seq data, and compare them with those of some other popular methods.

We discover that IF-PCA performs quite well in the aforementioned applications. Its success with microarray data was reported by Jin and Wang (2016), but it has never been applied to single-cell data. To use IF-PCA with single-cell data, we recommend a mild modification of the original procedure called IF-PCA(X), which performs the PCA step on the unnormalized data matrix *X* instead of the normalized data matrix *W*. On the eight single-cell RNA-seq data sets considered in this paper, IF-PCA(X) has the second-best accuracy in almost all the data sets, showing a stable performance across multiple data sets. We think IF-PCA has significant potential for single-cell clustering, as the method is simple, transparent, and tuning-free. Although the current IF-PCA(X) still underperforms compared to the state-of-the-art methods (e.g., SC3) in some data sets, it is hopeful that a variant of IF-PCA (say, by borrowing the consensus voting in SC3 or replacing PCA with some other embedding methods [Cai and Ma, 2022; Ma et al., 2023]) can outperform them.

We also find that UDL algorithms do not immediately yield improvements over classical methods with the microarray data and the single-cell data. The IF-VAE underperforms compared to IF-PCA in most data sets; there are only a few data sets in which the IF-VAE slightly outperforms IF-PCA. The reason can be either that non-linear dimension reduction has no significant advantage over linear dimension reduction in these data sets or IF-VAE is not optimally tuned. How to tune the deep learning algorithms in unsupervised settings is an interesting future research direction. Moreover, the theory on the VAE remains largely unknown (Fan et al., 2021). A theoretical investigation of the VAE requires an understanding of both the deep neural network structures and the variational inference procedure. We also leave this to future work.

The framework of IF-PCA only assumes feature sparsity but no other particular structures on the features. It is possible that the features are grouped (Chang et al., 2017) or have some tree structures (Li et al., 2021). How to adapt IF-PCA to this setting is an interesting yet open research direction.

In the real data analysis, we assume that the number of clusters, *K*, is given. When *K* is unknown, how to estimate *K* is a problem of independent interest. One approach is to use the scree plot. For example, Ke et al. (2023) proposed a method that first computes a threshold from the bulk eigenvalues in the scree plot and then applies this threshold to the top eigenvalues to estimate *K*. Another approach is based on global testing. Given a candidate *K*, we may first apply a clustering method with this given *K* and then apply the global testing methods in Jin et al. (2017) to test whether each estimated cluster has no sub-clusters; $\hat{K}$ is set as the smallest *K* such that the global null hypothesis is accepted in all estimated clusters. In general, estimating *K* is an independent problem from clustering. It is interesting to investigate which estimators of *K* work best for gene microarray data and single-cell RNA-seq data, which we leave to future work.

## Data Availability

Publicly available data sets were analyzed in this study. These data can be found here: https://data.mendeley.com/datasets/cdsz2ddv3t. https://data.mendeley.com/drafts/nv2x6kf5rd.
