# Supplementary material for: Subject clustering by IF-PCA and several recent methods
Source: Front Genet. 2023 May 23;14:1166404. doi: 10.3389/fgene.2023.1166404 (PMC10242062; doi:10.3389/fgene.2023.1166404)
Supplement: Supplementary file 1 [file DataSheet1.PDF]

Supplementary material for ‘Subject clustering by IF-PCA and several recent methods’

Dieyi Chen [1], **Jiashun Jin** [2*] **and Zheng Tracy Ke** [1]

[1] *Department of Statistics, Harvard University, Cambridge, MA, United States*
[2] *Department of Statistics, Carnegie Mellon University, Pittsburgh, PA, United States*

Correspondence*:
Jiashun Jin
jiashun@stat.cmu.edu

In this supplement, we prove all theoretical results in Section 4, namely, Theorems 4.1-4.3 and Lemma 4.1. Note that

- First, as mentioned before, the proof of Theorem 4.1 is long and similar to those in (Jin et al., 2017, Section 1.3) and is omitted.
- Second, to show Theorem 4.2, we apply Lemma 4.1 with $(N, m) = (n, p)$ to the X matrix in (1)-(4). Note that in our setting, for any fixed $0 < \theta < 1$ and $0 < \beta \leq 1/2$,

  $$\|\mu\|^2 \sim s_p \tau_p^2 \gg s_p(\tau_p^*)^2 = \sqrt{p/n} \geq \max(\sqrt{p/n}, \log(p+n)),$$

  so the conditions of Lemma 4.1 holds in this case, and Theorem 4.2 follows.
- Third, for Theorem 4.3, by elementary statistics, the event $\{\hat{S} \neq S(\mu)\}$ has a probability of $o(1)$. Therefore, the event only has a negligible effect over the Hamming error rate, so without loss of generality we can assume $\hat{S} = S(\mu)$. To show Theorem 4.3, we apply Lemma 4.1 to the matrix X_S, where $S = S(\mu)$ for short. Note that $|S(\mu) \sim \text{Bernoulli}(p, \epsilon_p)$ and concentrates at $s_p = p\epsilon_p$. For any fixed $0 < \theta < 1$ and $1/2 < \beta < 1$, by the definitions of τ_p^*,

  $$\|\mu_S\|^2 \sim s_p \tau_p^2 \geq s_p(\tau_p/\tau_p^*)^2 = s_p(\tau_p/\tau_p^*)^2 \geq (\tau_p/\tau_p^*)^2 \max\{s_p/\sqrt{n}, 1\}.$$

  By the condition of Theorem 4.3,

  $$\tau_p/(\sqrt{\log(p)}\tau_p^*) \to \infty.$$

  Therefore, with high probability,

  $$\|\mu_S\|^2 \gg \log(p) \max\{s_p/\sqrt{n}, 1\} \geq \max\{\sqrt{s_p/n}, \log(n + |S|)\},$$

  so the conditions of of Lemma 4.1 holds, and Theorem 4.3 follows.

Combining these comments, all remains to prove is Lemma 4.1.

0.1 Proof of Lemma 4.1

Recall that ξ is the first left-singular vector of $X \in \mathbb{R}^{N,m}$, $\|\mu\| \gg \max(\sqrt{m/N}, \log(m))$, and the goal is to show that as as $\min\{N, m\} \to \infty$, $\min\{\|\sqrt{N}\xi + Y\|_\infty, \|\sqrt{N}\xi - Y\|_\infty\} = o(1)$ with probability of $1 - O(m^{-3})$. To show this, we write

$$X = Y\mu' + Z, \qquad \text{where all } N \times m \text{ entries of } Z \text{ are iid } N(0, 1).$$

Let $H_0 = ZZ' - mI_N$. It is seen

$$XX' - mI_N = [\|\mu\|^2 YY' + Y\mu'Z' + Z\mu Y'] + ZZ' - mI_N = [\|\mu\|^2 YY' + Y\mu'Z' + Z\mu Y'] + H_0. \quad (0.1)$$

Let λ be the first eigenvalue of XX'. Since ξ is a left singular vector of X, $\lambda\xi = [\|\mu\|^2(\xi, Y) + (\xi, Z\mu)]Y + (\xi, Y)Z\mu + H_0\xi$. Rearranging it, we have

$$\sqrt{N}\xi = (I_N - (1/\lambda)H_0)^{-1}[b_1 Y + b_2 Z(\mu/\|\mu\|)], \quad (0.2)$$

where $b_1 = b_1(Y, Z, \mu) = (1/\lambda) \cdot [\sqrt{N}\|\mu\|^2(\xi, Y) + \sqrt{N}(\xi, Z\mu)]$ and $b_2 = b_2(Y, Z, \mu) = (1/\lambda)\sqrt{N}\|\mu\|(\xi, Y)$. Therefore, $\min\{\|\sqrt{N}\xi - Y\|_\infty, \|\sqrt{N}\xi + Y\|_\infty\}$ is no greater than

$$\min\{|b_1 - 1|, |b_1 + 1|\} + |b_1|\|Y - (I_N - (1/\lambda)H_0)^{-1}Y\|_\infty + |b_2|\|(I_N - (1/\lambda)H_0)^{-1}Z(\mu/\|\mu\|)\|_\infty. \quad (0.3)$$

To show the claim, it is sufficient to show that with probability at least $1 - o(m^{-3})$,

$$\min\{|b_1 - 1|, |b_1 + 1|\} = o(1), \qquad |b_2| = \frac{o(1)}{\sqrt{\log(m)}}, \quad (0.4)$$

and

$$\|Y - (I_N - (1/\lambda)H_0)^{-1}Y\|_\infty = o(1), \qquad \|(I_N - \frac{1}{\lambda}H_0)^{-1}Z(\mu/\|\mu\|)\|_\infty \leq C\sqrt{\log(m)}. \quad (0.5)$$

For later use, note that by Random Matrix Theory Vershynin (2012), with probability at least $1 - o(m^{-3})$,

$$\|H_0\| \leq C(N + \sqrt{Nm}), \qquad \|Z\| \leq C(\sqrt{N} + \sqrt{m}). \quad (0.6)$$

We now show (0.4). Consider the first item. Since Z and μ are independent, we have that with probability at least $1 - o(m^{-3})$, $|(\xi, Z\mu)| \leq \|\mu\| \cdot \|Z(\mu/\|\mu\|)\| \leq 2\|\mu\|\sqrt{N}$. Combining this with the triangle inequality,

$$\begin{aligned}
&\min\{b_1 - 1, b_1 + 1\} \\
\leq &(N\|\mu\|^2/\lambda)|\cos(Y, \xi) - 1| + |1 - (N\|\mu\|^2/\lambda)| + (\sqrt{N}/\lambda)|(\xi, Z\mu)| \\
\leq &(N\|\mu\|^2/\lambda)|\cos(Y, \xi) - 1| + |1 - (N\|\mu\|^2/\lambda)| + 2N\|\mu\|/\lambda, \quad (0.7)
\end{aligned}$$

where $\cos(Y, \xi) = \frac{(Y,\xi)}{\|Y\|\|\xi\|}$. At the same time, we rewrite (0.1) as

$$XX' - mI_N = A + H_0, \text{ where } A = \|\mu\|^2 YY' + Y\mu'Z' + Z\mu Y' \text{ for short.} \quad (0.8)$$

Note that A is a symmetric matrix of rank 2. For short, write $\nu = \|\mu\|^{-2}\mu$ and $a = a(Y, \mu, Z) = (1 + 4N^{-1}[Y'Z\nu + \|Z\nu\|^2])^{1/2}$. Let $\lambda_\pm$ be the two nonzero eigenvalues of A, and let $\eta_\pm$ be the corresponding eigenvectors. By elementary algebra,

$$\lambda_\pm(A) = N\|\mu\|^2[(1/2)(1 \pm a) + N^{-1}Y'Z\nu], \qquad \eta_\pm \propto (1/2)(1 \pm a)Y + Z\nu. \tag{0.9}$$

By elementary statistics, $Y'Z\nu \sim N(0, N/\|\mu\|^2) \sim (\sqrt{N}\|\mu\|^{-1})N(0, 1)$. By Mills' ratio of $N(0, 1)$ (e.g., Durrett (2005)), we have that with probability at least $1 - o(m^{-3})$, $|Y'Z\nu| \leq C\sqrt{\log(m)}\sqrt{N}\|\mu\|^{-1}$. Similarly, $\|Z\nu\|^2 \leq C\sqrt{\log(m)}N\|\mu\|^{-2}$. Therefore, it is seen that with probability at least $1 - o(m^{-3})$, $N^{-1}[|Y'Z\nu| + \|Z\nu\|^2]$ does not exceed

$$C\sqrt{\log(m)}N^{-1}[(\sqrt{N}\|\mu\|^{-1}) + N\|\mu\|^{-2}] = C\sqrt{\log(m)}[(\sqrt{N}\|\mu\|)^{-1} + \|\mu\|^{-2}]. \tag{0.10}$$

From the condition of the current lemma, we have

$$\|\mu\|^2 \gg \max(\sqrt{m/N}, \log(m)). \tag{0.11}$$

Inserting it into (0.10) gives that $N^{-1}[|Y'Z\nu| + \|Z\nu\|^2] = o(1)$. Plugging this into the expression of the term a, it follows that with probability at least $1 - o(m^{-3})$, $|a - 1| = o(1)$. Combining this with (0.9),

$$|(N\|\mu\|^2/\lambda_+) - 1| = o(1), \qquad (\lambda_-/\lambda_+) = o(1) \qquad |\cos(Y, \eta_+) - 1| = o(1). \tag{0.12}$$

At the same time, by (0.6), $\|H_0\| = \|ZZ' - mI_m\| \leq C(n + \sqrt{mN})$. Combining these with (0.11)-(0.12) gives

$$\|(1/\lambda_+)H_0\| \leq C(N + \sqrt{mN})/(N\|\mu\|^2) = o(1). \tag{0.13}$$

This says that in (0.8), the leading eigenvalue of A is larger than that of H_0. By Weyl's Inequality (Bai and Silverstein, 2010), we have that $|\lambda_+ - \lambda| \leq \|H_0\|$. By the Sine-theta theorem (Davis and Kahan, 1970) , we know $\cos(\eta_+, \xi) \geq 1 - \|H_0\|/\lambda_1(A)$. Therefore, Combining (0.12) and (0.13), with probability at least $1 - o(m^{-3})$,

$$|\lambda_+/\lambda - 1| \leq \|H_0\|/\lambda = o(1), \qquad |\cos(\eta_+, \xi) - 1| = o(1). \tag{0.14}$$

Combining (0.12) and (0.14) gives

$$|(N\|\mu\|^2/\lambda) - 1| = o(1), \qquad |\cos(Y, \xi) - 1| = o(1). \tag{0.15}$$

In particular, combining (0.11), (0.13), and (0.14) gives that with probability at least $1 - o(m^{-3})$,

$$\|(1/\lambda)H_0\| = o(1), \qquad \sqrt{mN}/\lambda = o(1). \tag{0.16}$$

Inserting (0.15) and (0.11) into (0.7) gives the first item of (0.4).

Consider the second item of (0.4). Note that $|b_2| \leq (N\|\mu\|/\lambda)$, where by (0.15), the right hand side $\leq \|\mu\|^{-1}$. The claim follows directly from (0.11).

Next, we show (0.5). Since the proofs are similar, we only show the first item. Let e_1 be the first base vector of R^n. Note that by symmetry, and by using the union bound and triangle inequality, it is sufficient

to show that with probability at least $1 - o(m^{-4})$,

$$\left|e_1'(I_N - \frac{1}{\lambda}H_0)^{-1}e_1 - 1\right| = o(1), \qquad |e_1'(I_N - \frac{1}{\lambda}H_0)^{-1}(Y - Y_1e_1)| \leq C\sqrt{\log(m)}. \qquad (0.17)$$

Consider the first item of (0.17). To show the first claim, by basic algebra $\left|e_1'(I_N - \frac{1}{\lambda}H_0)^{-1}e_1 - 1\right| \leq \|(I_N - \frac{1}{\lambda}H_0)^{-1} - I_N\| \leq \|(I_N - \frac{1}{\lambda}H_0)^{-1}\|\|(I_N - \frac{1}{\lambda}H_0) - I_N\|\|I_N\|$ with $\|I_N\| = 1$ and $\|\frac{1}{\lambda}H_0\| = o(1)$ by (0.16). By Weyl's Inequality (Bai and Silverstein, 2010), we have that $\lambda_{\min}(I_N - \frac{1}{\lambda}H_0) \geq \lambda_{\min}(I_N) - \|\frac{1}{\lambda}H_0\| \geq 1 - o(1) \geq 0.5$, thus $\|(I_N - \frac{1}{\lambda}H_0)^{-1}\| = \lambda_{\min}^{-1}(I_N - \frac{1}{\lambda}H_0) \leq 2$. This proves the first item of (0.17).

Consider the second item in (0.17). Write $Y = (Y_1, \tilde{Y})'$, and let $\tilde{Z}$ be the $(N-1) \times m$ matrix consisting all but the first row of Z, and let $\tilde{H}_0 = \tilde{Z}\tilde{Z}' - mI_{N-1}$. It follows that

$$I_N - (1/\lambda)H_0 = \begin{pmatrix} 1 - (1/\lambda)[\|Z_1\|^2 - m], & -(1/\lambda)Z_1'\tilde{Z} \\ -(1/\lambda)\tilde{Z}Z_1, & I_{N-1} - (1/\lambda)\tilde{H}_0 \end{pmatrix}.$$

By matrix inversion formula (e.g., Horn and Johnson (1985)),

$$e_1'(I_N - \frac{1}{\lambda}H_0)^{-1}(Y - Y_1e_1) = (e_1'[I_N - (1/\lambda)H_0]^{-1}e_1) \cdot (1/\lambda)Z_1'\tilde{Z}'[I_{N-1} - (1/\lambda)\tilde{H}_0]^{-1}\tilde{Y}. \quad (0.18)$$

Now, since rows of Z are independent, Z_1 and $\tilde{Z}[I_{N-1} - (1/\lambda)\tilde{H}_0]^{-1}\tilde{Y}$ are two vectors that almost independent of each other; the only issue is that Z_1 is correlated with λ. To overcome the difficulty, we write

$$\frac{Z_1'\tilde{Z}'[I_{N-1} - (1/\lambda)\tilde{H}_0]^{-1}\tilde{Y}}{\lambda} = \sum_{k=0}^{\infty}\frac{Z_1'\tilde{Z}\tilde{H}_0^k\tilde{Y}}{\lambda^{k+1}} = \sum_{k=0}^{\infty}\frac{\|\tilde{Z}\tilde{H}_0^k\tilde{Y}\|}{\lambda^{k+1}} \cdot \frac{Z_1'\tilde{Z}\tilde{H}_0^k\tilde{Y}}{\|\tilde{Z}\tilde{H}_0^k\tilde{Y}\|}. \qquad (0.19)$$

Now, for each k, Z_1 and $\tilde{Z}\tilde{H}_0^k\tilde{Y}$ are independent, and so

$$Z_1'(\tilde{Z}'\tilde{H}_0^k\tilde{Y}/\|\tilde{Z}'\tilde{H}_0^k\tilde{Y}\|) \sim N(0,1).$$

For the k-th term on the RHS of (0.19), with probability $1 - o(m^{-4(k+1)})$, there is $|Z_1'(\tilde{Z}'\tilde{H}_0^k\tilde{Y}/\|\tilde{Z}'\tilde{H}_0^k\tilde{Y}\|)| \leq \sqrt{8(k+1)\log(m)}$. Considering all k, $\sum_{k=0}^{\infty} o(m^{-4(k+1)}) = o(m^{-4})$. Therefore, with probability at least $1 - o(m^{-4})$,

$$(1/\lambda)^{k+1}\|\tilde{Z}\tilde{H}_0^k\tilde{Y}\| \leq \sqrt{(N-1)}(1/\lambda)\|\tilde{Z}\|\left(\frac{\|\tilde{H}_0\|}{\lambda}\right)^k.$$

Combining these with (0.19) and (0.16), it is seen that with probability at least $1 - o(m^{-4})$,

$$\begin{aligned}|(1/\lambda)Z_1'\tilde{Z}'[I_{N-1} - (1/\lambda)\tilde{H}_0]^{-1}\tilde{Y}| &\leq \sqrt{(N-1)}(1/\lambda)\|\tilde{Z}\|\sqrt{8\log(m)}\sum_{k=0}^{\infty}\left[\left(\frac{\|\tilde{H}_0\|}{\lambda}\right)^k\sqrt{k+1}\right] \\ &\leq C\sqrt{(N-1)}(1/\lambda)\|\tilde{Z}\|\sqrt{8\log(m)}.\end{aligned}$$

Inserting this into (0.18) and combining with the first item of (0.17), the first item of (0.15), and (0.11), the second item of (0.17) follows. This proves (0.5) and completes the proof of Lemma 4.1.

REFERENCES

Bai, Z. and Silverstein, J. (2010). *Spectral Analysis of Large Dimensional Random Matrices* (Springer)

Davis, C. and Kahan, W. M. (1970). The rotation of eigenvectors by a perturbation. iii. *SIAM Journal on Numerical Analysis* 7, 1–46

Durrett, R. (2005). *Probability: Theory and Examples (3rd Edition)* (Brooks/Cole-Thomson Learning)

Horn, R. and Johnson, C. (1985). *Matrix Analysis* (Cambridge University Press)

Jin, J., Ke, Z. T., and Wang, W. (2017). Phase transitions for high dimensional clustering and related problems. *Annals of Statistics* 45

Vershynin, R. (2012). Introduction to the non-asymptotic analysis of random matrices. *Compressed Sensing* , 210–268
